# Supplementary material for: 3D atomic-scale imaging of mixed Co-Fe spinel oxide nanoparticles during oxygen evolution reaction
Source: Nat Commun. 2022 Jan 10;13:179. doi: 10.1038/s41467-021-27788-2 (PMC8748757; doi:10.1038/s41467-021-27788-2)
Supplement: Supplementary file 1 — Supplementary Information [file 41467_2021_27788_MOESM1_ESM.pdf]

# Supporting Information of 3-D atomic-scale imaging of mixed Co-Fe oxide nanoparticles during oxygen evolution reaction

Weikai Xiang[1], Nating Yang[2], Xiaopeng Li[3], Julia Linnemann[4], Ulrich Hagemann[5], Olaf Ruediger[6], Markus Heidelmann[5], Tobias Falk[7], Matteo Aramini[8], Serena DeBeer[6], Martin Muhler[7], Kristina Tschulik[4], Tong Li[1]*

*[1]Institute for Materials, Ruhr-Universität Bochum, Universitätsstraße 150, 44801 Bochum, Germany*

*[2]CAS Key Laboratory of Low-Carbon Conversion Science and Engineering, Shanghai Advanced Research Institute (SARI), Chinese Academy of Sciences (CAS), Shanghai 201210, China*

*[3]State Key Laboratory for Modification of Chemical Fibers and Polymer Materials and College of Materials Science and Engineering, Donghua University, Shanghai 201620, China*

*[4]Faculty of Chemistry and Biochemistry, Analytical Chemistry II, Ruhr-Universität Bochum, Universitätsstraße 150, 44801 Bochum, Germany*

*[5]Interdisciplinary Center for Analytics on the Nanoscale (ICAN) and Center for Nanointegration Duisburg-Essen (CENIDE), University of Duisburg-Essen, Carl-Benz-Straße 199, 47057 Duisburg, Germany*

*[6]Max Planck Institute for Chemical Energy Conversion, Stiftstraße 34-36, 45470 Mülheim an der Ruhr, Germany*

*[7]Faculty of Chemistry and Biochemistry, Laboratory of Industrial Chemistry, Ruhr-Universität Bochum, Universitätsstraße 150, 44801 Bochum, Germany*

*[8]Diamond Light Source, Harwell Science and Innovation Campus, Chilton, Didcot, OX11 0DE, UK*

*Corresponding author

E-mail: tong.li@rub.de

Tel: +49 (0)234 32 26099

Postal address: Universitätsstr.150, Bochum, 44801, Germany

**Keywords**: Atom probe tomography, electrochemistry, water splitting, catalyst degradation, spinel oxides

**Supplementary Figures**


Supplementary Fig. 1 XRD of pristine $Co_2FeO_4$ and $CoFe_2O_4$ 3
Supplementary Fig. 2 TEM images and SAED patterns of $Co_2FeO_4$ 4
Supplementary Fig. 3 TEM images and SAED patterns of $CoFe_2O_4$ 5
Supplementary Fig. 4 $N_2$ isotherms of pristine $Co_2FeO_4$ and $CoFe_2O_4$ 6
Supplementary Fig. 5 Electrochemical data normalised to electrode geometric surface area 7
Supplementary Fig. 6 Peak fitting and quantitative analysis of XPS data 8
Supplementary Fig. 7 XANES and XAS of $Co_2FeO_4$ and $CoFe_2O_4$ before and after 1000 cycles 9
Supplementary Fig. 8 HRTEM image of pristine $Co_2FeO_4$ 10
Supplementary Fig. 9 Additional HRTEM images of $Co_2FeO_4$ and $CoFe_2O_4$ after OER 11
Supplementary Fig. 10 HRTEM and EDX mapping of $Co_2FeO_4$ and $CoFe_2O_4$ after 500 cycles 12
Supplementary Fig. 11 APT specimen preparation 13
Supplementary Fig. 12 APT data of $Co_2FeO_4$ before and after OER 14
Supplementary Fig. 13 Mass spectra of pristine $Co_2FeO_4$ 15
Supplementary Fig. 14 Cross-sectional atom maps of pristine $Co_2FeO_4$ 16
Supplementary Fig. 15 1D concentration profiles and 1D profiles of counts of $Co_2FeO_4$ 17
Supplementary Fig. 16 Additional 1D concentration profiles of segregated $Co_2FeO_4$ 18
Supplementary Fig. 17 1D concentration profiles of non-segregated $Co_2FeO_4$ 19
Supplementary Fig. 18 Additional 1D concentration profiles of non-segregated $Co_2FeO_4$ 20
Supplementary Fig. 19 APT data of $CoFe_2O_4$ before and after OER 21
Supplementary Fig. 20 1D concentration profiles of $CoFe_2O_4$ 22
Supplementary Fig. 21 Additional 1D concentration profiles of $CoFe_2O_4$ 23
Supplementary Fig. 22 Effect of laser energy on oxide stoichiometry measurement 24
Supplementary Fig. 23 $H_2$ TPR profiles of CuO and pristine $Co_2FeO_4$ 25
Supplementary Fig. 24 Equivalent circuit models 26
Supplementary Fig. 25 Capacitances and resistances of $Co_2FeO_4$ and $CoFe_2O_4$ during OER 26


**Supplementary Tables**


Supplementary Table 1. Structure and lattice constants of oxides 27
Supplementary Table 2. Number of nanoparticles analysed by APT 27
Supplementary Table 3. Number of $Co^{II}$ in tetragonal sites in $Co_2FeO_4$ and $CoFe_2O_4$ 28


**Supplementary Notes**


Supplementary Note 1 XAS 29
Supplementary Note 2 APT specimen preparation 29
Supplementary Note 3 Measurement of oxide stoichiometry by APT and H2 TPR 29-31
Supplementary Note 4 EIS 31-32
**Supplementary References** 33

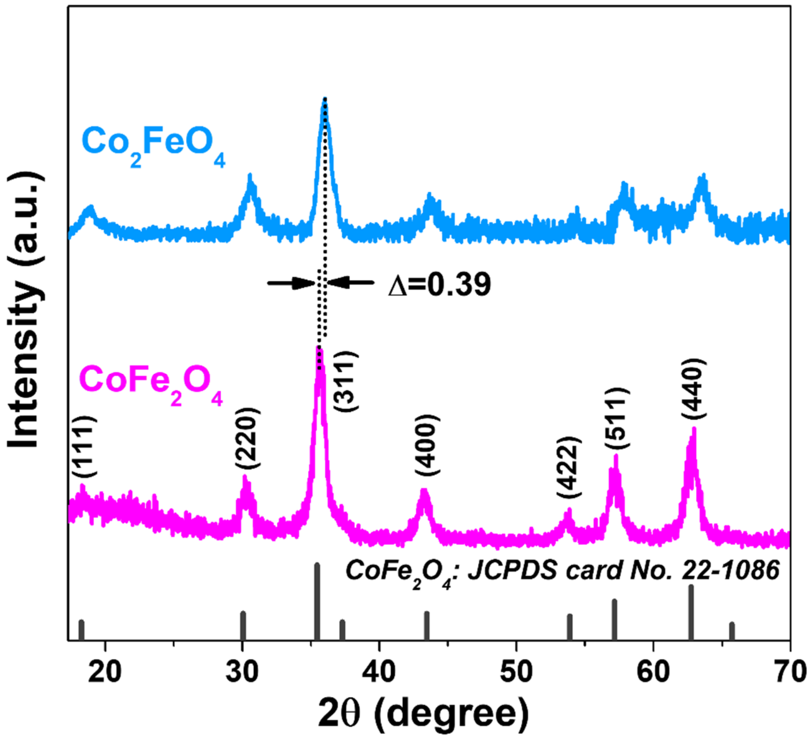


**Supplementary Fig. 1 Powder X-ray diffraction (XRD) of the pristine $CoFe_2O_4$ and $Co_2FeO_4$ nanoparticles.** The XRD diffraction peaks reveal that both Co-Fe oxides match well with the spinel structure (JCPDS card No: 22-1086). The 2θ of lattice plane (311) for $Co_2FeO_4$ slightly shifts to a higher angle in comparison with the value for the $CoFe_2O_4$ sample since the ionic radius of $Fe^{3+}$ (0.65 Å) is larger than that of $Co^{2+/3+}$ (0.61 Å)[1].

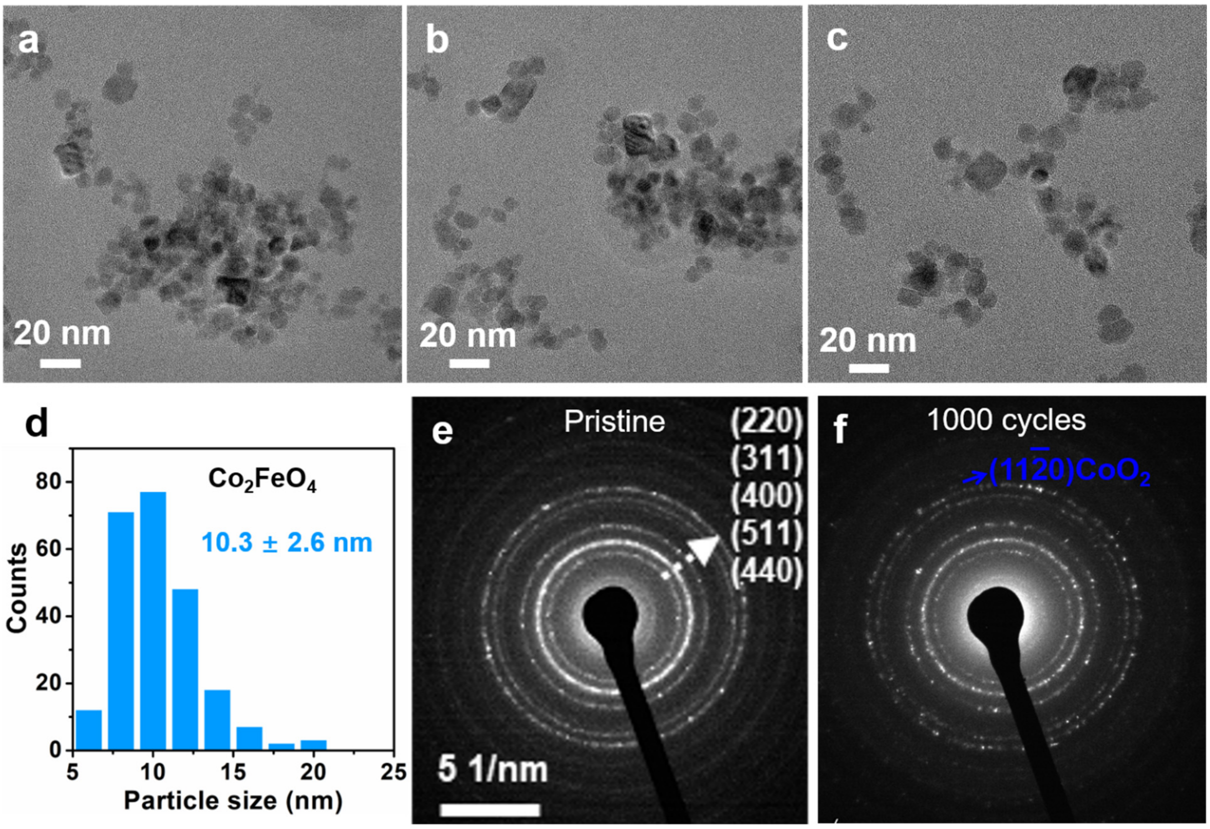


**Supplementary Fig. 2 TEM images and selected area electron diffraction (SAED) patterns of $Co_2FeO_4$.** (**a-c**) TEM images of the pristine $Co_2FeO_4$ nanoparticles, (**d**) size histogram measured from (a-d) indicating that the size of $Co_2FeO_4$ is 10.3 ± 2.6 nm, (**e**) SAED of pristine $Co_2FeO_4$ nanoparticles (the diffraction rings corresponding to the lattice planes (220), (311), (400), (511) and (440)) and (**f**) SAED of $Co_2FeO_4$ nanoparticles after 1000 cycles showing additional reflection spots that correspond to $(11\bar{2}0)CoO_2$. The error bar in (d) is the standard deviation calculated from $\sqrt{\frac{\sum_{i=1}^{n}(x_i-\bar{x})^2}{n-1}}$ where $\bar{x}$ is the mean nanoparticles size and n is the total number of nanoparticles.

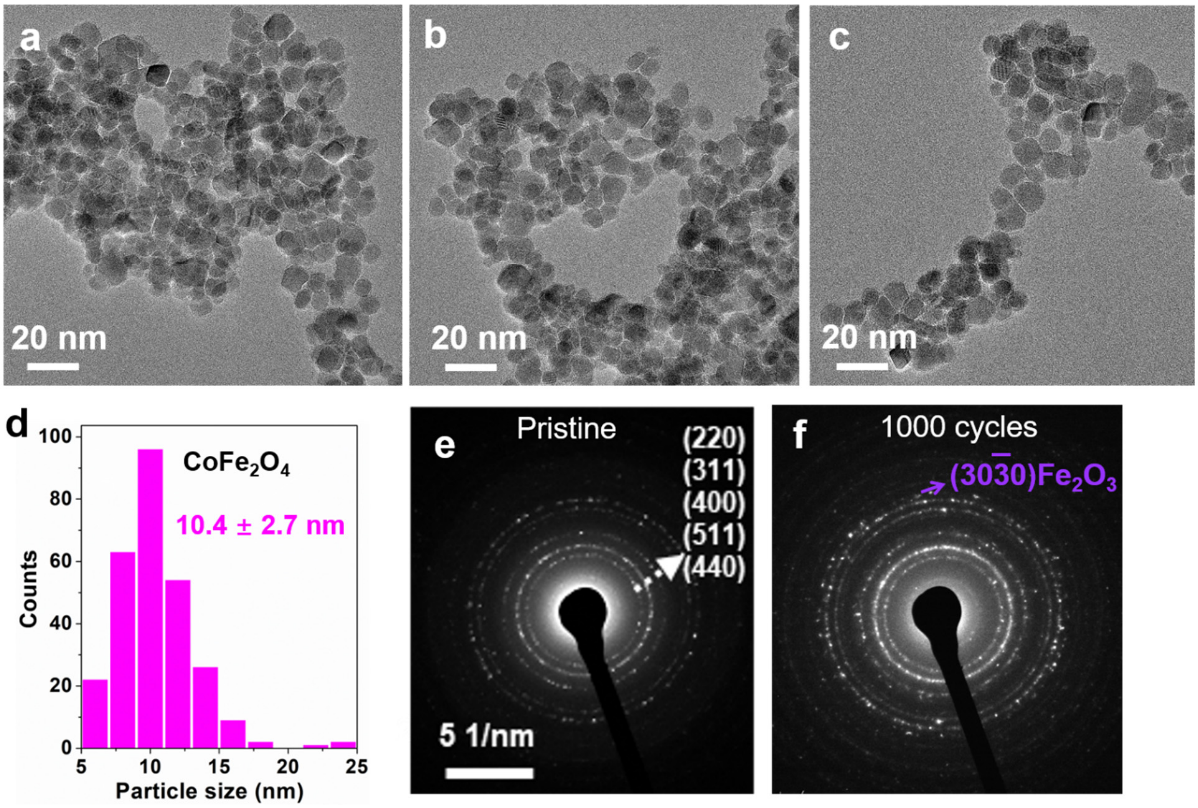


**Supplementary Fig. 3 TEM images and SAED patterns of $CoFe_2O_4$.** (**a-c**) TEM images of the pristine $CoFe_2O_4$ nanoparticles, (**d**) size histogram showing that the size of $Co_2FeO_4$ is 10.4 ± 2.7 nm, (**e**) SAED pattern of pristine $CoFe_2O_4$ nanoparticles (the diffraction rings corresponding to the lattice planes (220), (311), (400), (511) and (440)) and (**f**) SAED pattern of $CoFe_2O_4$ nanoparticles after 1000 cycles of cyclic voltammetry measurements showing additional reflection spots corresponding to $(30\bar{3}0)Fe_2O_3$. The error bar in (d) is the standard deviation calculated from $\sqrt{\frac{\sum_{i=1}^{n}(x_i-\bar{x})^2}{n-1}}$ where $\bar{x}$ is the mean nanoparticles size and n is the total number of nanoparticles.

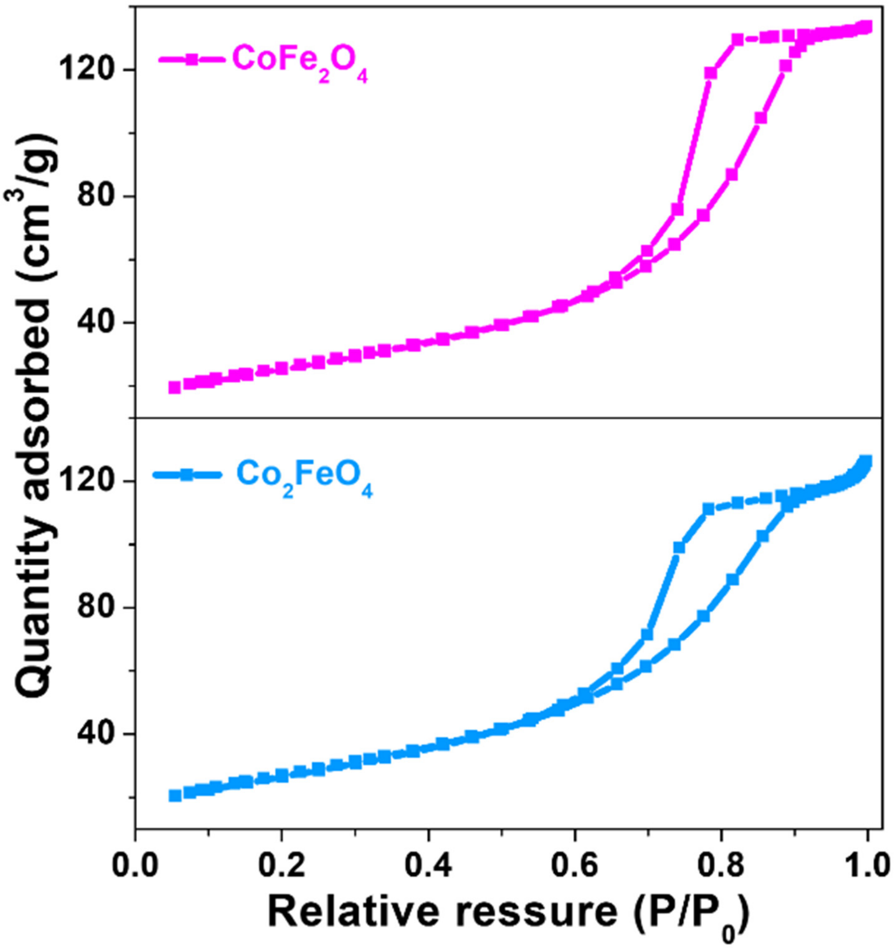


**Supplementary Fig. 4 $N_2$ isotherms of pristine $Co_2FeO_4$ and $CoFe_2O_4$.** The specific surface areas were derived to be 93 and 98 $m^2/g$ for $CoFe_2O_4$ and $Co_2FeO_4$, respectively, according to the BET equation.

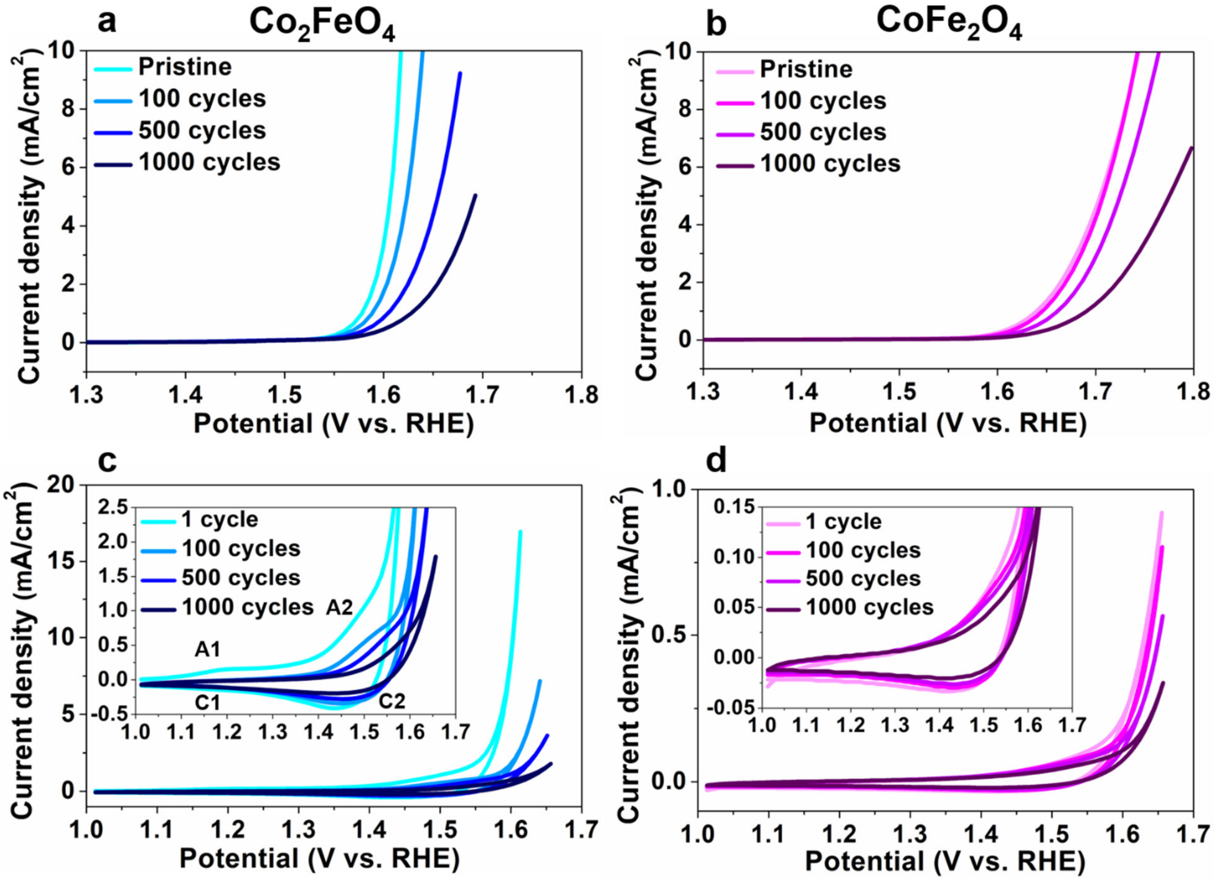


**Supplementary Fig. 5 Electrochemical data of $Co_2FeO_4$ and $CoFe_2O_4$ normalised to geometric surface area of glassy carbon.** (**a**, **b**) Linear sweep voltammetry and (**c**, **d**) cyclic voltammetry data of $CoFe_2O_4$ and $Co_2FeO_4$ shown in Figs. 1a-d with current density normalised to the geometric surface area of the glassy carbon electrodes.

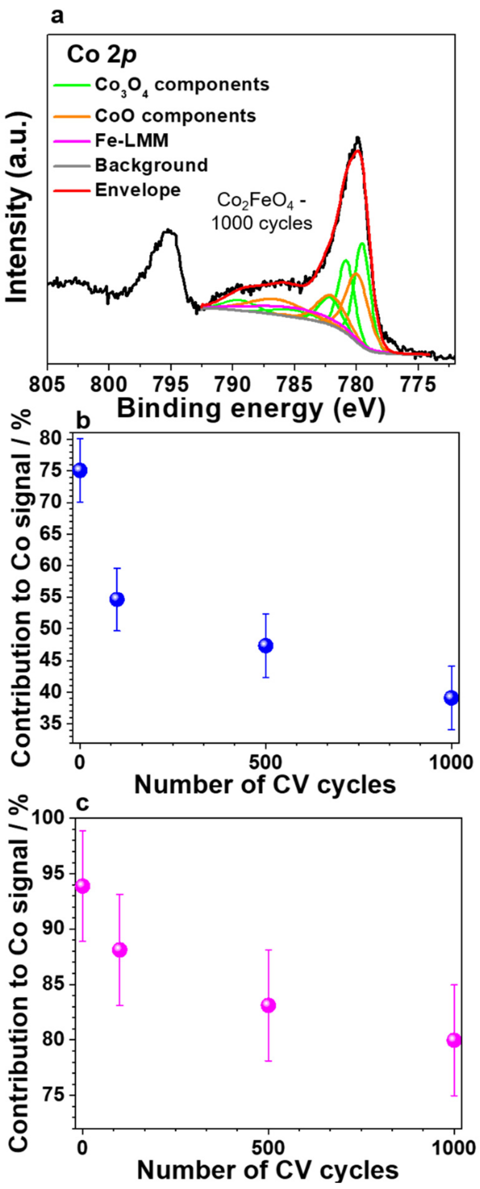


**Supplementary Fig. 6 Peak fitting and quantitative analysis of XPS data. (a)** an example of peak fitting of Co 2$p_{3/2}$ spectrum of $Co_2FeO_4$ after 1000 cycles of OER (various oxides and hydroxides have been attempted to fit the Co-2*p* spectra found and the combination of CoO- and $Co_3O_4$-like Co has yielded the best results. The overlap of Fe-LMM peak with the Co-2*p* signal when using Al-Ka light has been taken into account by measuring Iron-oxides reference samples and including the peak shape and intensities into the Co spectrum and vice versa, as the Co-LMM overlaps with the Fe 2*p* signal). The contribution of CoO-like Co(II) to Co 2$p_{3/2}$ signal of (**b**) $Co_2FeO_4$ and (**c**) $CoFe_2O_4$ decreases as the number of CV cycles increase, while opposite trend was observed for the contribution of $Co_3O_4$-like Co (not shown in the plot). The proportion of Co(II) of $Co_2FeO_4$ decreases from ~75% in the pristine state to ~40% after 1000 cycles. In contrast, the proportion of Co(II) of $CoFe_2O_4$ is ~95% and decreased to 80% after 1000 cycles. The error bars are the peak fitting uncertainties derived from varying the starting parameters of the fit and the background limits.

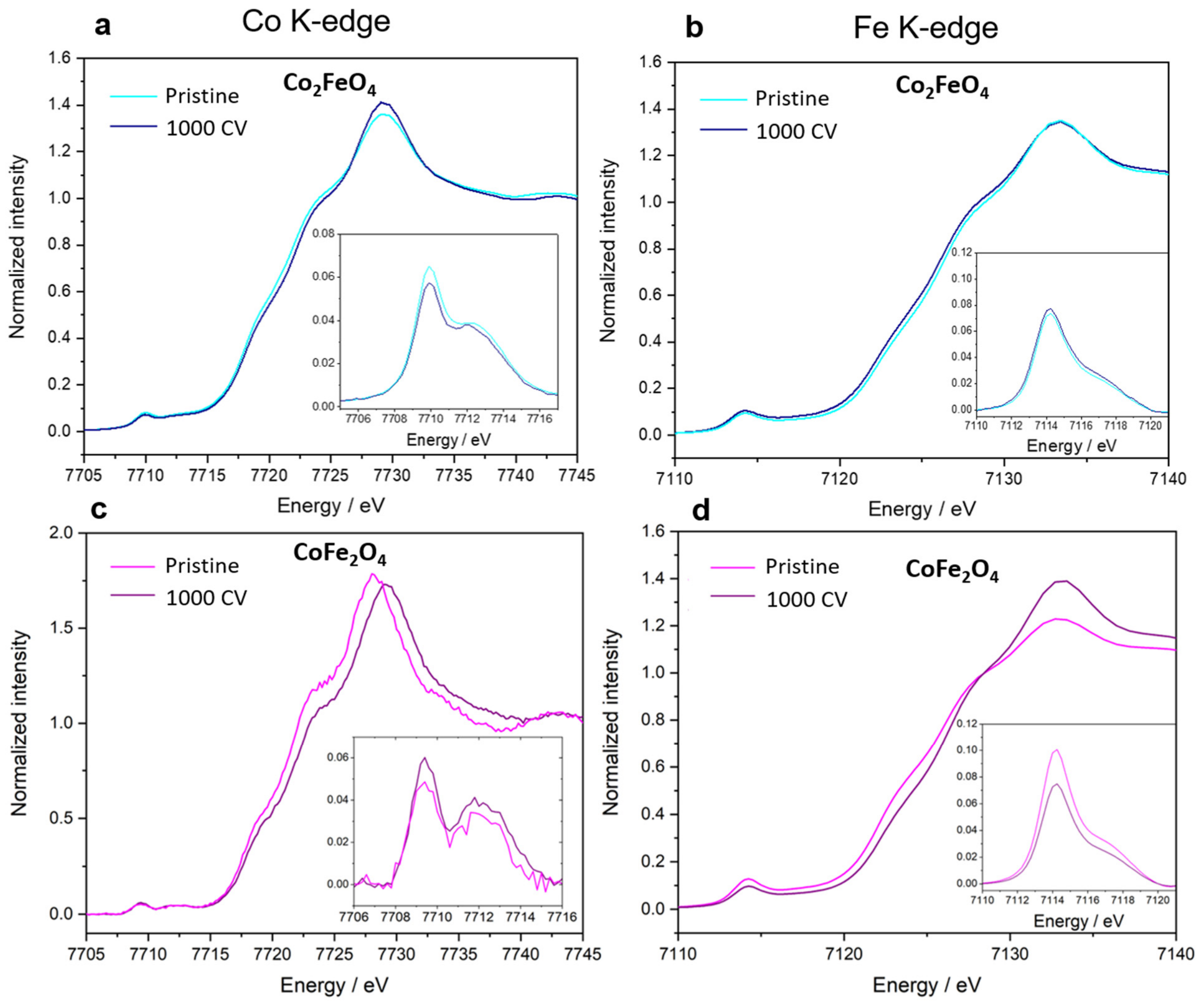


**Supplementary Fig. 7 XANES and XAS of $Co_2FeO_4$ and $CoFe_2O_4$ before and after 1000 cycles.** (**a-b**) Co and Fe K-edge XANES spectra of $Co_2FeO_4$ in the pristine state and after the 1000$^{th}$ cycle. (**c**) Co Kβ-detected HERFD XAS spectra and (**d**) Fe XAS spectra of $CoFe_2O_4$ in the pristine state and after the 1000$^{th}$ cycle (the insets show the baseline-corrected pre-edge region in detail). Detailed description is listed in Supplementary Note 1.

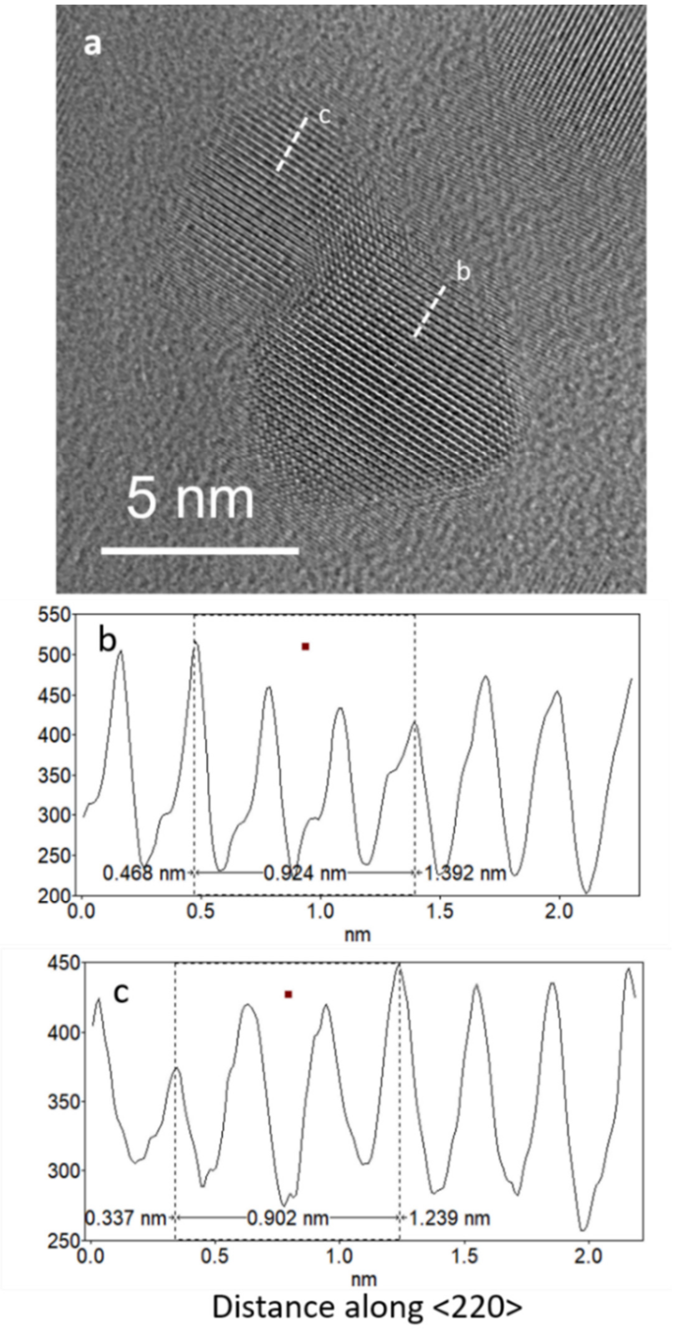


**Supplementary Fig. 8 HRTEM image of pristine $Co_2FeO_4$**. (**b-c**) Intensity profiles obtained along the dashed lines in HRTEM image in (**a**) showing the difference of $d_{220}$ interplanar spacing on the left and right side of the $Co_2FeO_4$ nanoparticle. The intensity profiles in two regions of a $Co_2FeO_4$ nanoparticle along the <220> direction shows that the interplanar spacing of $d_{220}$ is approx. 0.308 nm and 0.301 nm on the left and right part of the $Co_2FeO_4$ nanoparticle, respectively. Such difference possibly originates from a slight deviation of Fe content in these two regions (as indicated by the APT data in Fig. 4).

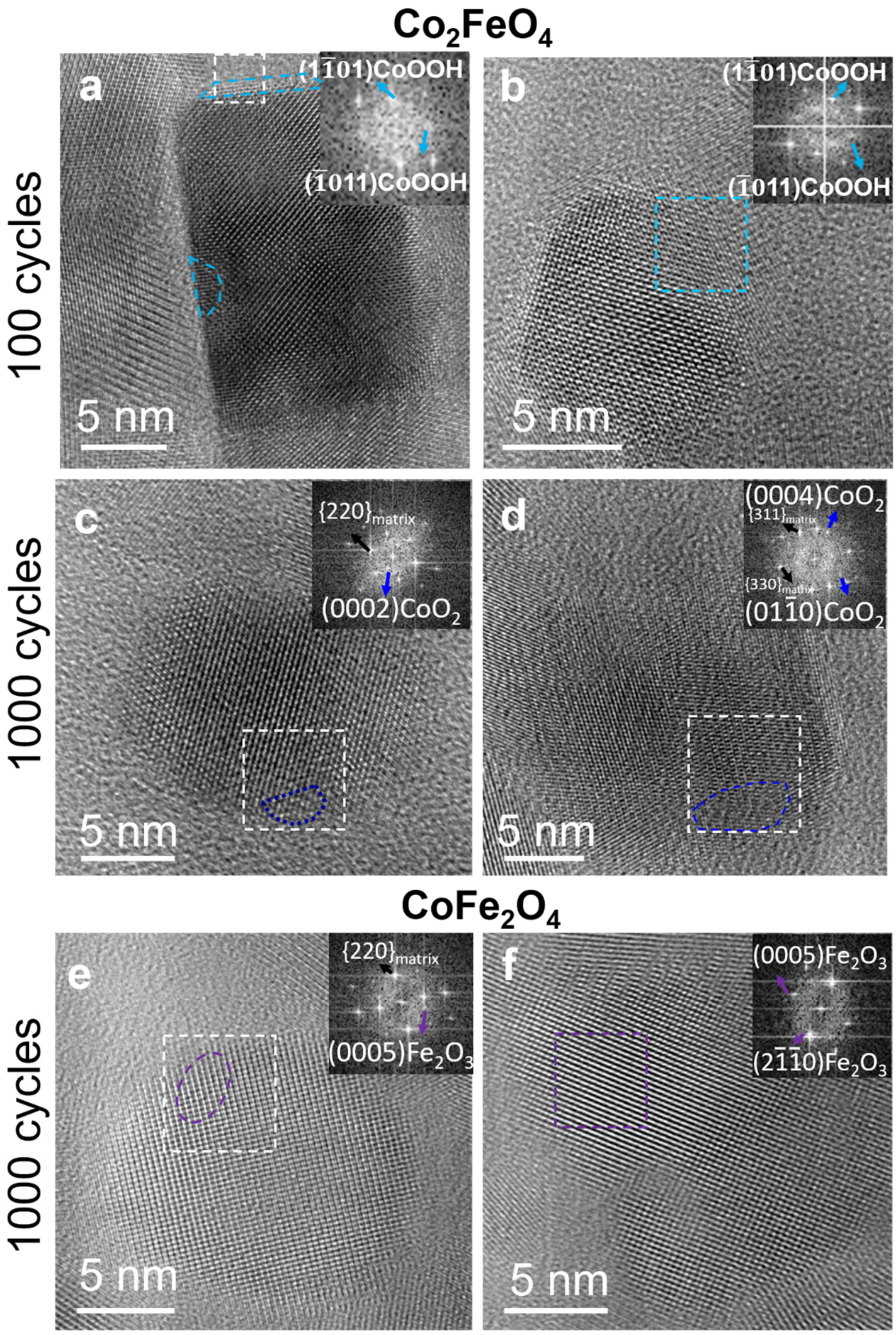


**Supplementary Fig. 9 Additional HRTEM images of $Co_2FeO_4$ and $CoFe_2O_4$ after OER.** HRTEM images of $Co_2FeO_4$ after (**a-b**) 100 cycles and (**c-d**) 1000 cycles showing the possible presence of β-CoOOH (marked by light-blue-dotted areas) and $CoO_2$ (dark-blue-dotted areas) after 100 and 1000 cycles respectively, and (**e-f**) $CoFe_2O_4$ after 1000 cycles suggesting the formation of $Fe_2O_3$ (purple-dotted regions). The Fourier filtered transform images (insets) were obtained from the dashed square regions.

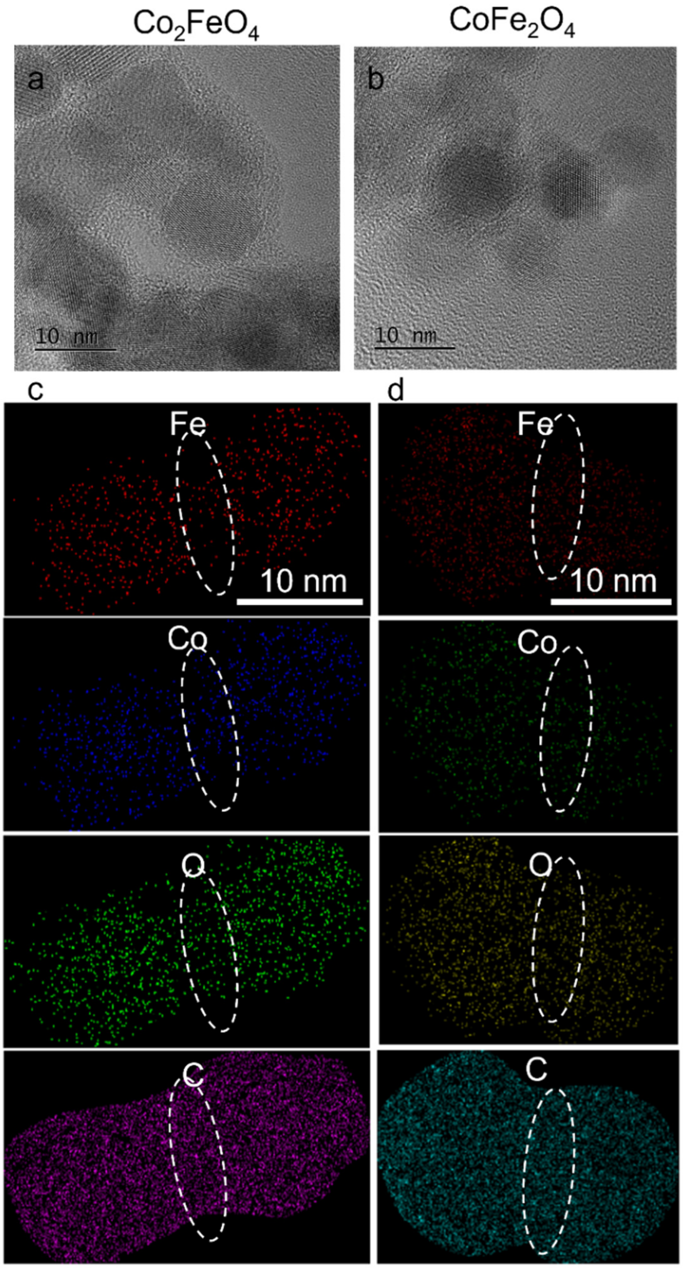


**Supplementary Fig. 10** HRTEM image and EDX mapping of (**a,c**) $Co_2FeO_4$ and (**b,d**) $CoFe_2O_4$ after 500 cycles. After 500 cycles, amorphous layers are observed on the surface of $Co_2FeO_4$ and $CoFe_2O_4$ nanoparticles (Supplementary Figs. 10a-b). To confirm the composition of the amorphous layer, we performed the TEM/EDX mapping (Supplementary Figs. 10c-d). The surfaces and regions where two nanoparticles are closely connected are not enriched with Co, Fe, but with C, Supplementary Figs. 10c-d. We conclude that the amorphous layer is not comprised of the active CoOOH, as has been reported previously[2]. Instead, this amorphous layer is the result of electron beam induced carbon deposition, where hydrocarbons were adsorbed on the samples after electrochemical measurements or during sample transfer.

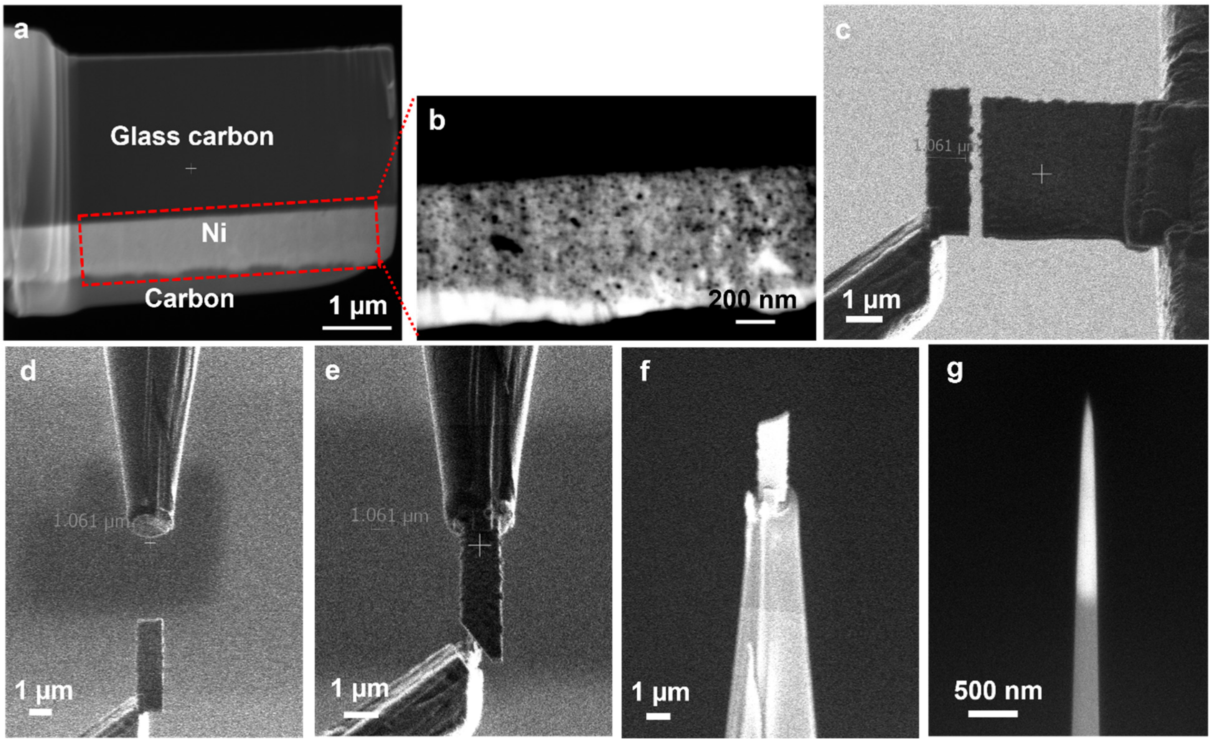

**Supplementary Fig. 11 APT specimen preparation.** (**a**) SEM image of a TEM lamella of glassy carbon deposited by a layer of Ni, (**b**) backscattered electron image of the bottom section of the TEM lamella (highlighted by the dashed box in a) showing the presence of the nanoparticles, (**c**) a thin slice cut from the TEM lamella, lifted out by the manipulator, (**d**) landed on a Si microtip, (**e-f**) detached from the manipulator and (**g**) final annular milling till the tip was sharpened less than 100 nm in diameter. More details are provided in Supplementary Note 2.

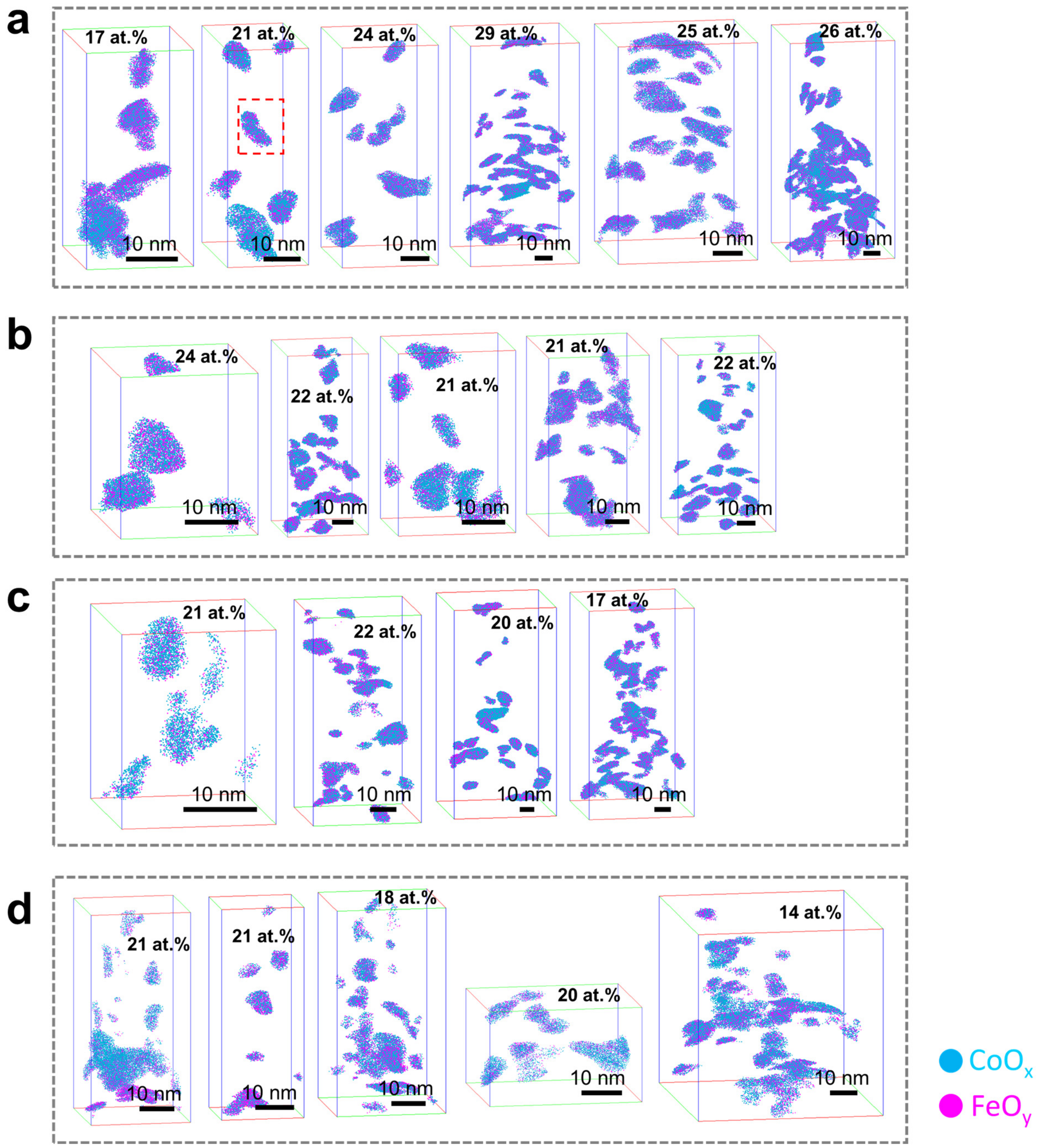


**Supplementary Fig. 12 APT data of $Co_2FeO_4$ before and after OER.** 3D-APT reconstructions of (**a**) pristine $Co_2FeO_4$ nanoparticles, the nanoparticles after (**b**) 100 cycles, (**c**) 500 cycles, and (**d**) 1000 cycles of CV measurements under the OER conditions (the Ni matrix not shown), obtained by exporting the data within the (Co+Fe) iso-concentration surface at various values indicated above each APT reconstructions (the reason why the iso-concentration surface values vary across different datasets is that the compositions of nanoparticles vary, as indicated by the composition histograms shown in Figs. 4m-n and Figs. 5m-n).

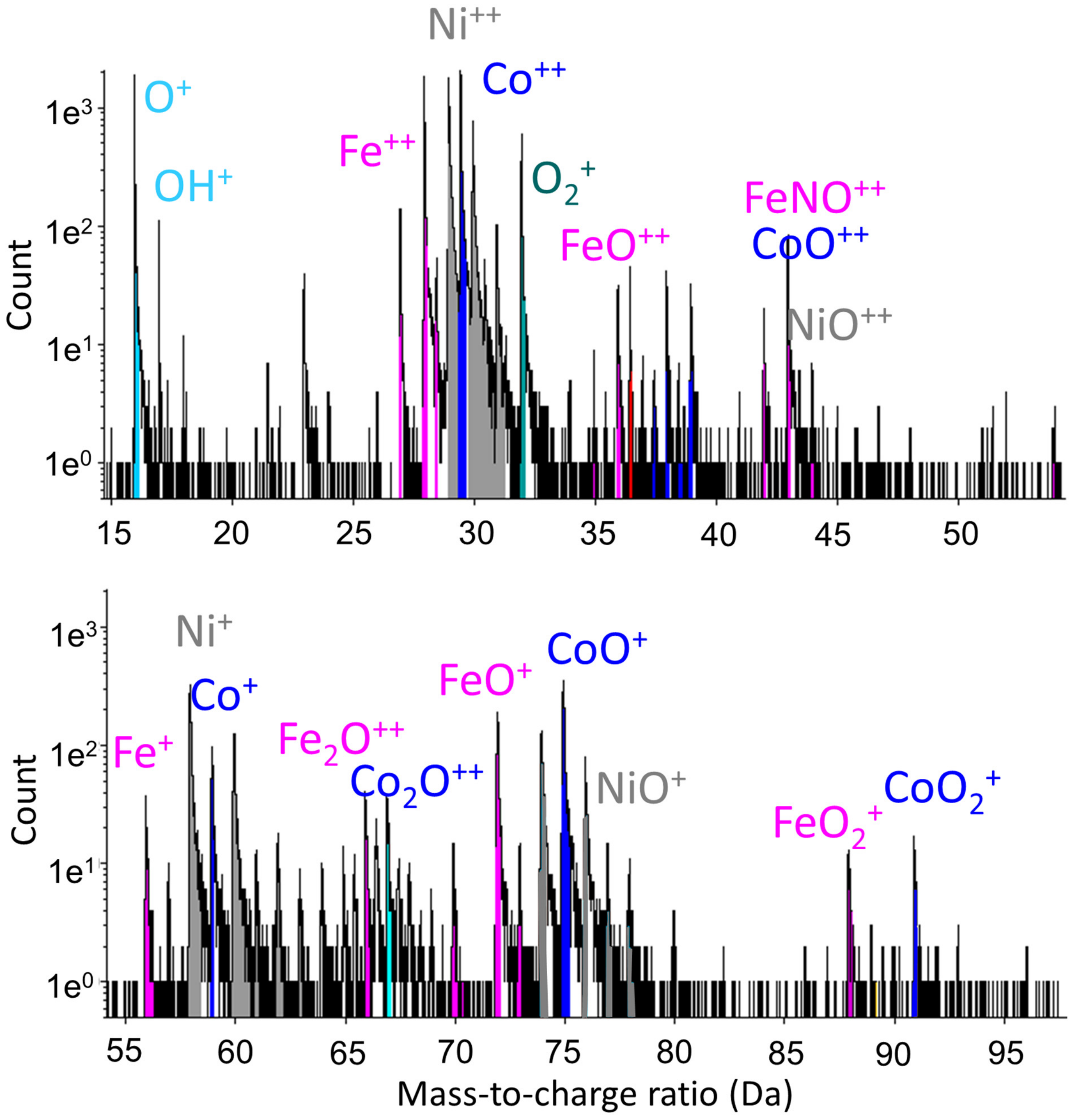


**Supplementary Fig. 13 Mass spectrum of pristine $Co_2FeO_4$**. It shows that the laser-assisted field evaporation of the oxides produces O, Co, Fe and molecular FeO, FeNO (possibly the salts from synthesis), $Fe_2O$, $FeO_2$, CoO, $Co_2O$ and $CoO_2$ along with Ni and NiO ions from the Ni matrix.

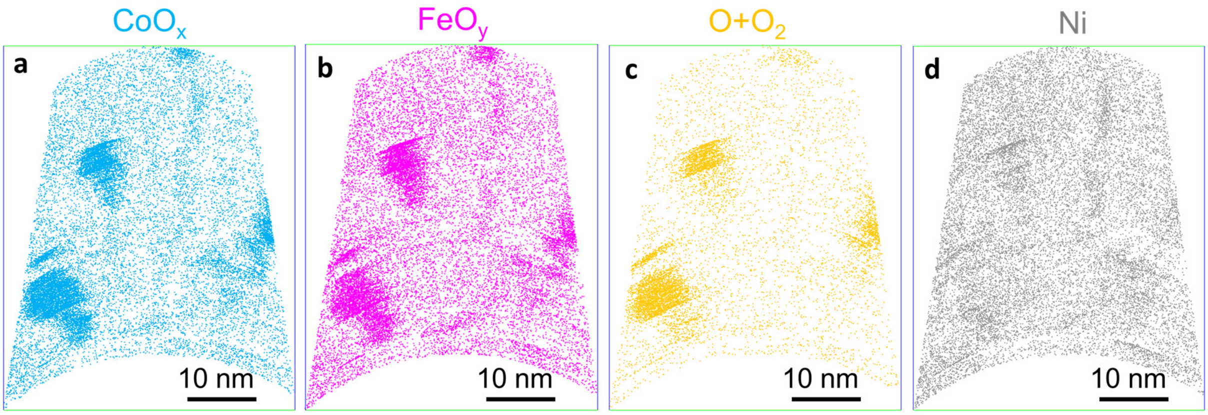


**Supplementary Fig. 14 Cross-sectional atom maps of pristine $Co_2FeO_4$.** APT reconstructions of pristine $Co_2FeO_4$ showing the distribution of (**a**) $CoO_x$, (**b**) $FeO_y$, (**c**) O and (**d**) Ni ionic species (the same APT reconstruction shown in Fig. 4a). The $CoO_x$ atom map includes Co, $Co_2O$, CoO and $CoO_2$ complex molecular ions. The $FeO_y$ atom map contains Fe, $Fe_2O$, FeO, $FeO_2$ complex molecular ions. The O atom map is comprised of O and $O_2$ complex ions. (Note that the complex molecular ions are the fragments of $Co_2FeO_4$ during the field laser-assisted field evaporation.)

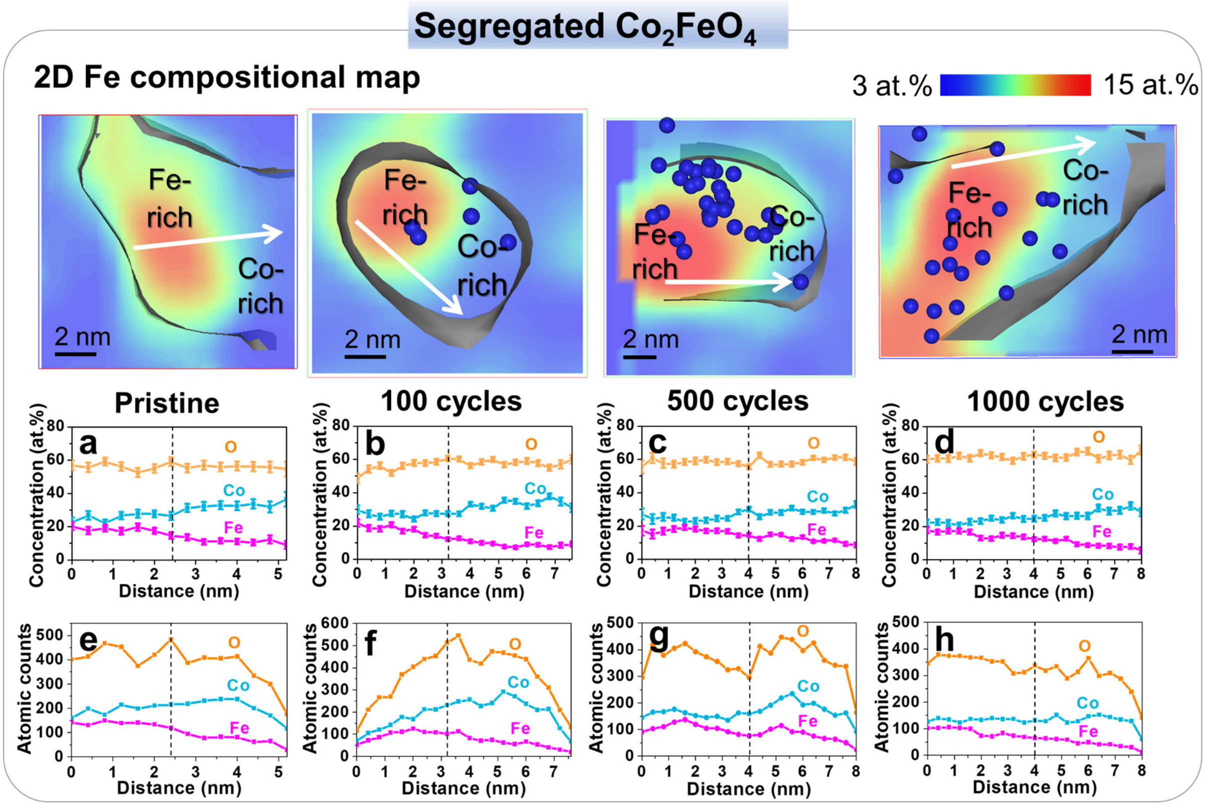


**Supplementary Fig. 15 1D concentration profiles and 1D profiles of counts of $Co_2FeO_4$.** (**a-d**) 1D concentration profiles, obtained by placing analysis cylinders along the white arrows marked in the 2D Fe compositional map of segregated $Co_2FeO_4$ (shown in Fig. 4i-l), indicating the composition of the Fe-rich and Co-rich nanodomains (the obtained ratios of Co/Fe and O/M from these concentration profiles along with values obtained from other nanoparticles summarised in Fig. 4m-n and the ratios calculated from the total counts listed in Tables 1-2), (**e-h**) atomic counts of the 1D profiles in (a-d) showing that the number of Co counts is nearly the same in both Co-rich and Fe-rich nanodomains. In contrast, the Fe counts in the Co-rich nanodomain are less than those in the Fe-rich nanodomains. This result suggests the increased Co/Fe ratio (with increasing CV cycles) is not caused by Co enrichment, instead the Fe dissolution. The error bars for the concentration were calculated from $\sqrt{\frac{c(100-c)}{N}}$, where c is the concentration (in at.%) and N is the total number of atoms within a bin of the profile.

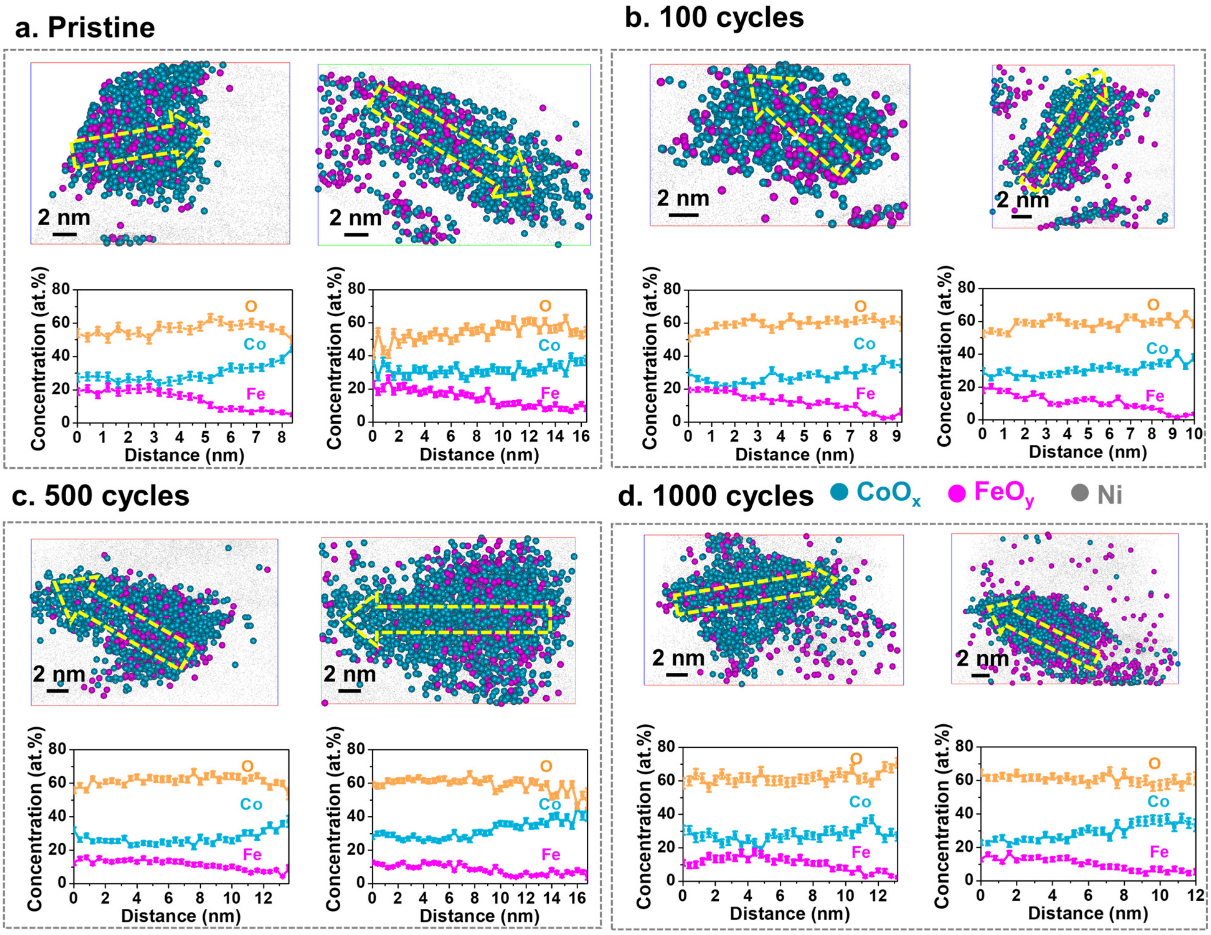


**Supplementary Fig. 16 Additional two examples of segregated $Co_2FeO_4$ nanoparticles.** APT reconstructions and corresponding 1D concentration profiles of segregated $Co_2FeO_4$ nanoparticles in the (**a**) pristine state and after (**b**) 100 cycles, (**c**) 500 cycles and (**d**) 1000 cycles along with 1D concentration profiles plotted along the yellow arrows. The error bars for the concentration were calculated from $\sqrt{\frac{c(100-c)}{N}}$, where c is the concentration (in at.%) and N is the total number of atoms within a bin of the profile.

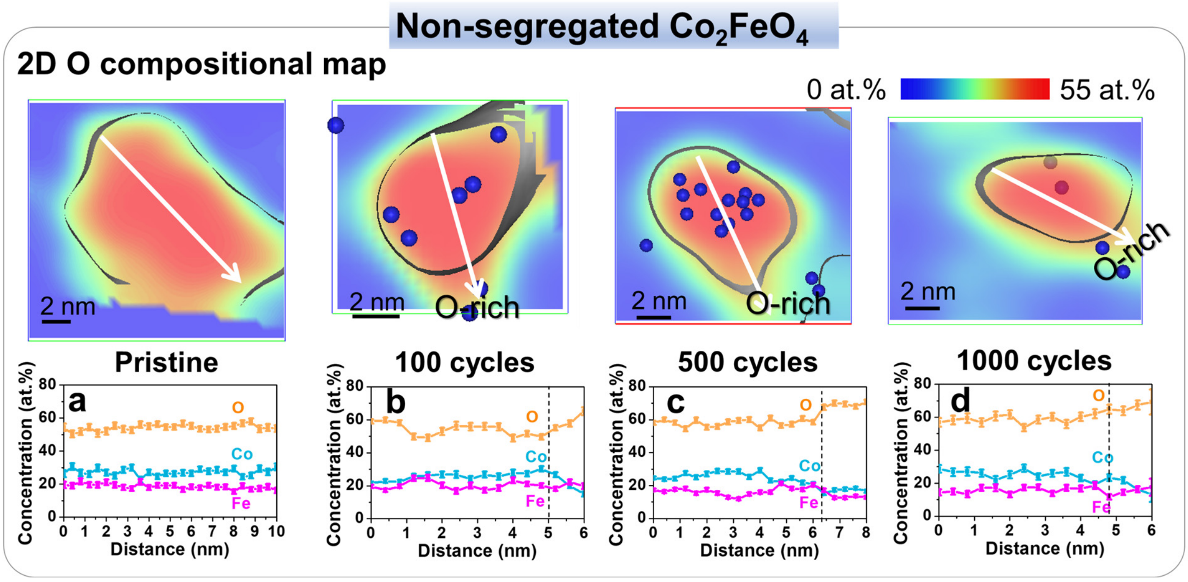


**Supplementary Fig. 17 1D concentration profiles of non-segregated $Co_2FeO_4$.** (a-d) 1D concentration profiles obtained along the white arrows marked in the 2D O compositional map of non-segregated $Co_2FeO_4$ shown in Fig. 5i-l indicating the composition in the oxygen-rich regions and bulk part (the obtained ratios of Co/Fe and O/M along with values from other nanoparticles summarised in Fig. 5m-n and the ratios calculated from the total counts listed in Tables 1-2). The error bars for the concentration were calculated from $\sqrt{\frac{c(100-c)}{N}}$, where c is the concentration (in at.%) and N is the total number of atoms within a bin of the profile.

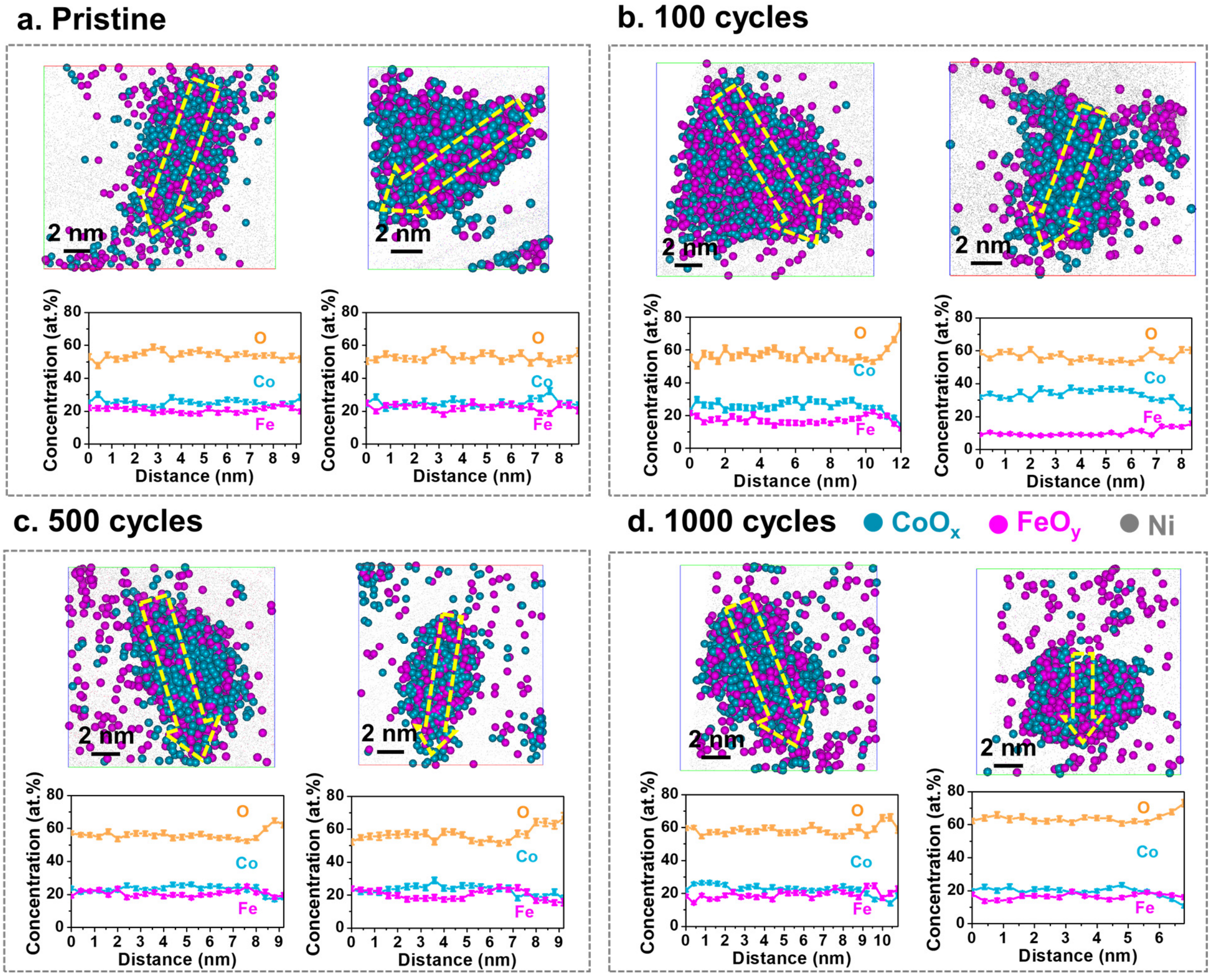


**Supplementary Fig. 18 Additional two examples of non-segregated $Co_2FeO_4$ nanoparticles.** APT reconstructions and corresponding 1D concentration profiles of non-segregated $Co_2FeO_4$ nanoparticles in the (**a**) pristine state and after (**b**) 100 cycles, (**c**) 500 cycles and (**d**) 1000 cycles along with 1D concentration profiles plotted along the yellow arrows. The error bars for the concentration were calculated from $\sqrt{\frac{c(100-c)}{N}}$, where c is the concentration (in at.%) and N is the total number of atoms within a bin of the profile.

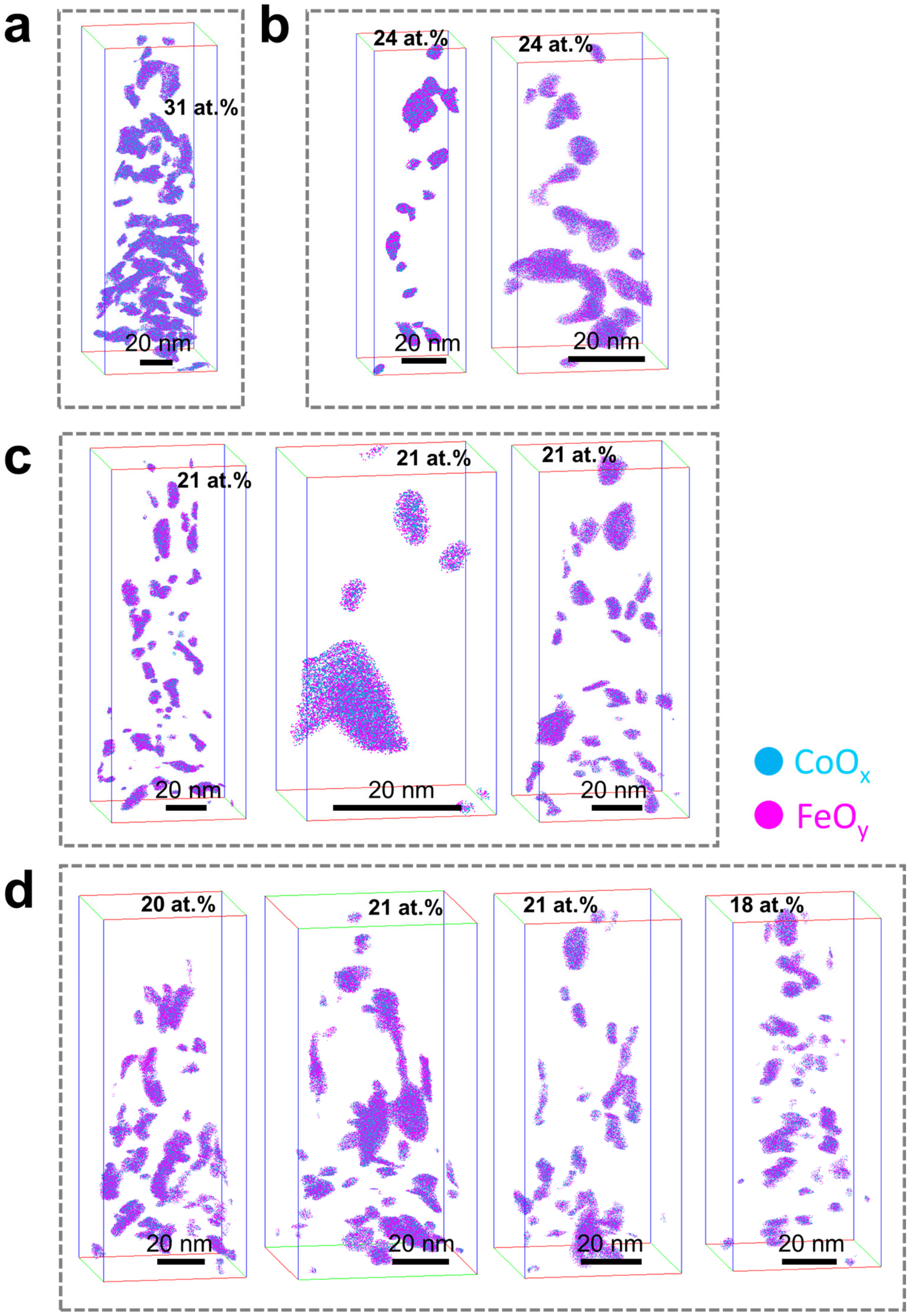


**Supplementary Fig. 19 APT data of $CoFe_2O_4$ before and after OER.** 3D-APT reconstructions of (**a**) pristine $CoFe_2O_4$ nanoparticles, the nanoparticles after (**b**) 100 cycles, (**c**) 500 cycles, and (**d**) 1000 cycles (the Ni matrix not shown), obtained by exporting the data within the (Co+Fe) iso-concentration surface at various values indicated above each APT reconstruction.

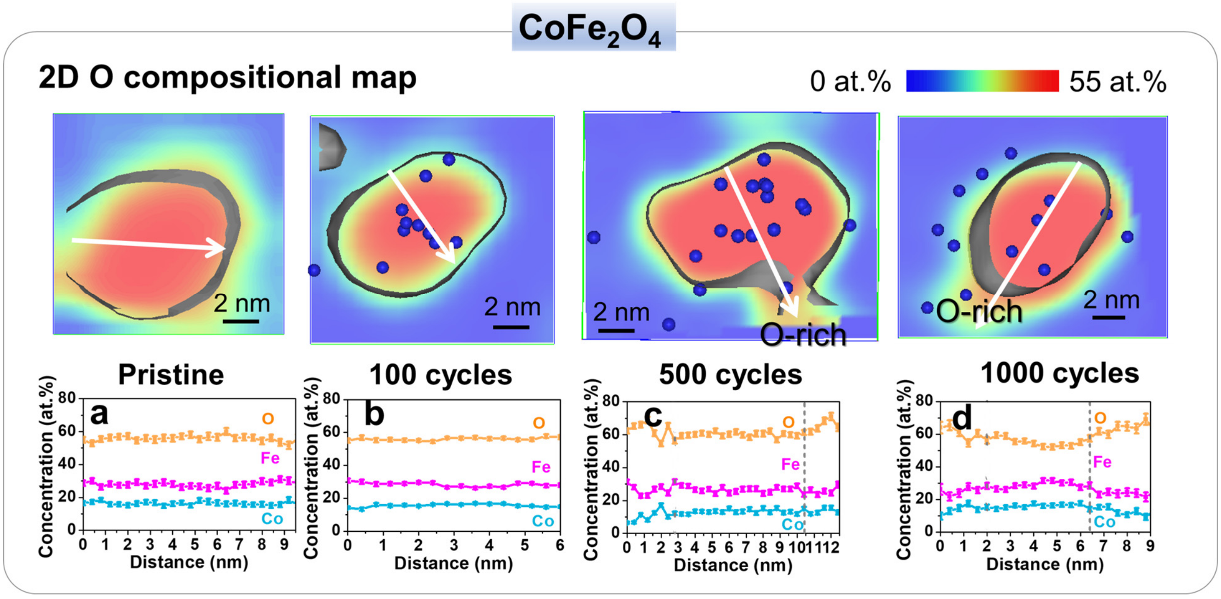


**Supplementary Fig. 20 1D concentration profiles of $CoFe_2O_4$.** (**a-d**) 1D concentration profiles obtained along the white arrows marked in the 2D O compositional map of $CoFe_2O_4$ shown in Fig. 6i-l showing the composition in the oxygen-rich regions and bulk part (the obtained ratios of Co/Fe and O/M along with values from other nanoparticles summarised in Fig. 6m-n and the ratios calculated from the total counts listed in Tables 1-2). The error bars for the concentration were calculated from $\sqrt{\frac{c(100-c)}{N}}$, where c is the concentration (in at.%) and N is the total number of atoms within a bin of the profile.

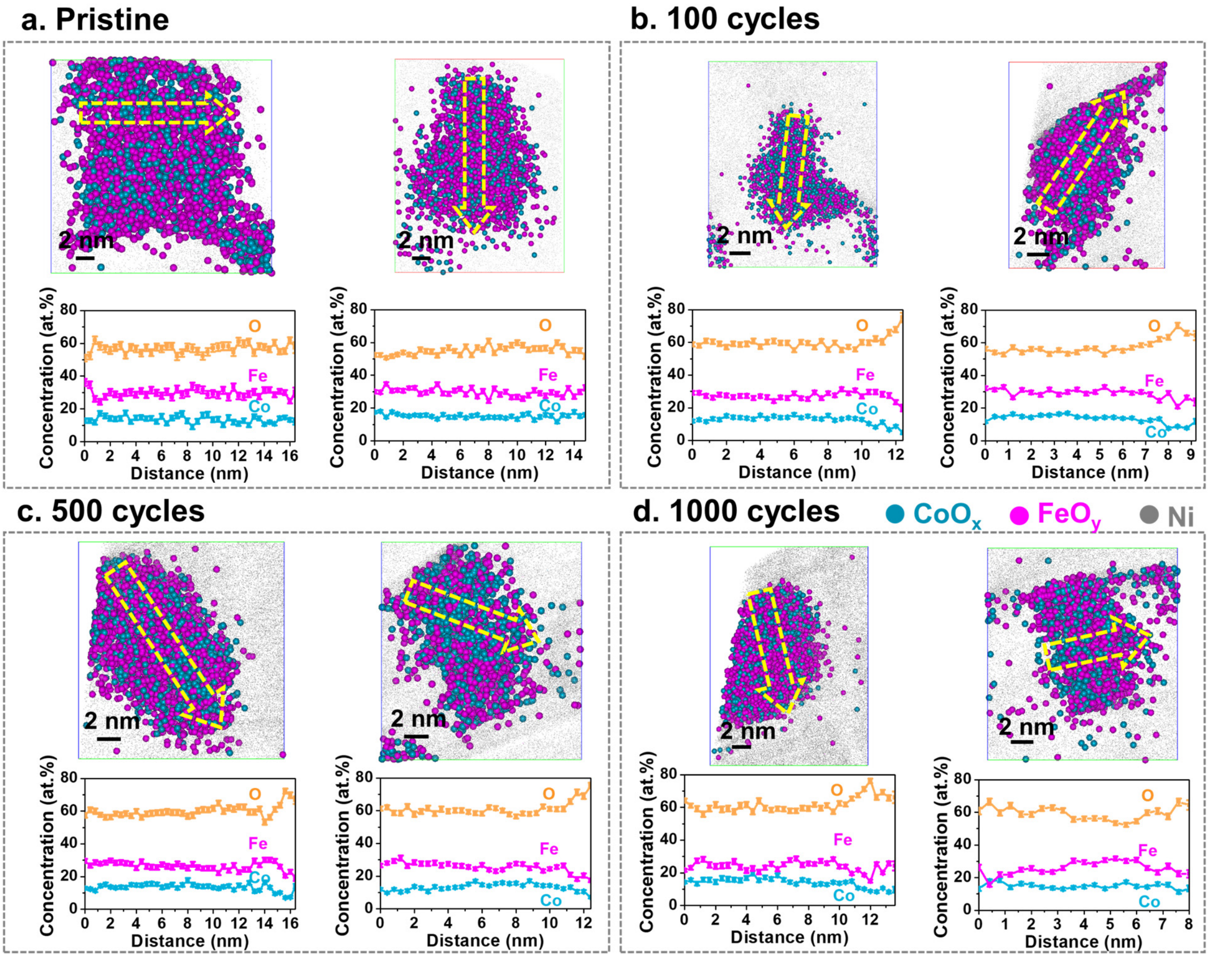


**Supplementary Fig. 21 Additional two examples of $CoFe_2O_4$ nanoparticles.** APT reconstructions and corresponding 1D concentration profiles of $CoFe_2O_4$ nanoparticles in the (**a**) pristine state and after (**b**) 100 cycles, (**c**) 500 cycles and (**d**) 1000 cycles along with 1D concentration profiles plotted along the yellow arrows. The error bars for the concentration were calculated from $\sqrt{\frac{c(100-c)}{N}}$, where c is the concentration (in at.%) and N is the total number of atoms within a bin of the profile.

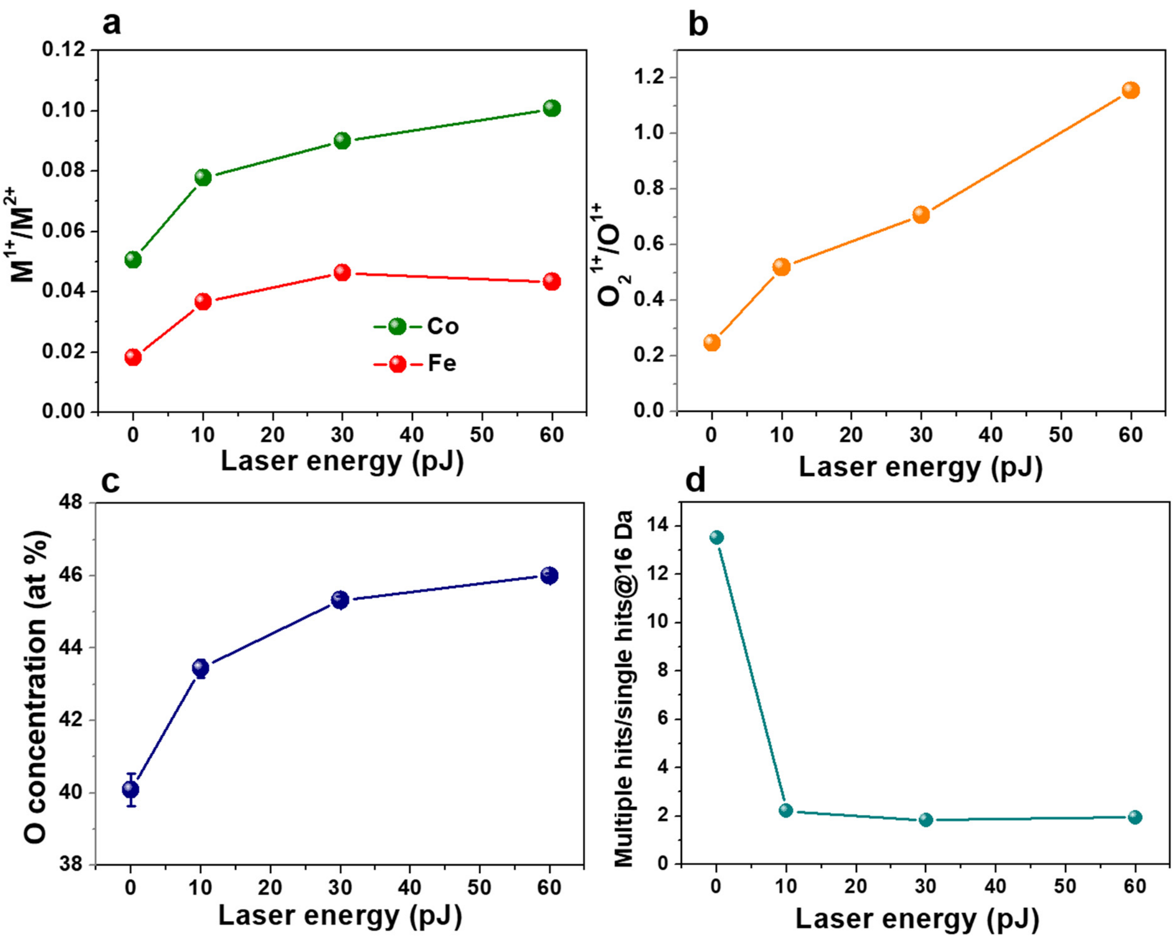


**Supplementary Fig. 22 Effect of laser energy on the oxide stoichiometry measurement of pristine $Co_2FeO_4$ nanoparticles embedded in Ni matrix**. (**a**) $M^{1+}/M^{2+}$ ratio, (**b**) $O_2^{1+}/O^{1+}$ ratio, (**c**) O concentration, and (**d**) the ratio of multiple hits to single hits at 16 Da measured from APT data of pristine $Co_2FeO_4$ versus laser energy. Detailed discussion can be found in Supplementary Note 3.

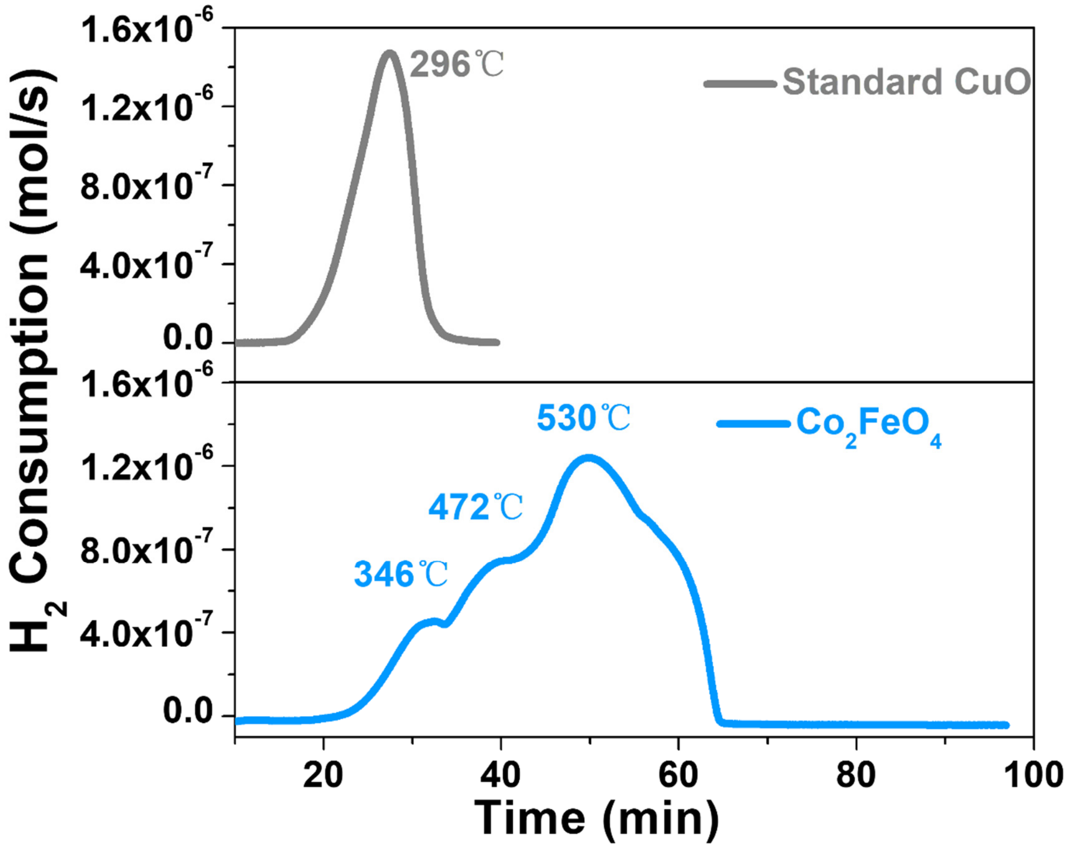


**Supplementary Fig. 23 $H_2$ TPR profiles of standard CuO and the pristine $Co_2FeO_4$ and nanoparticles.**

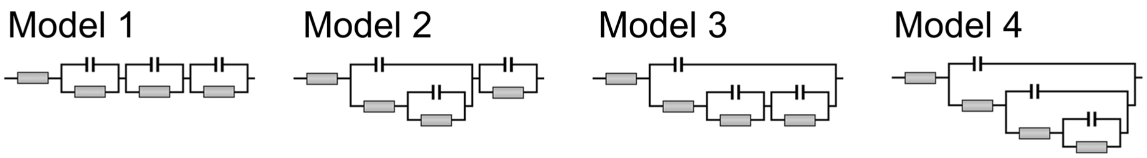


**Supplementary Fig. 24 Equivalent circuit models**. They comprise a series resistance and three resistor and capacitor elements, which were applied to analyse the EIS data of $Co_2FeO_4$.

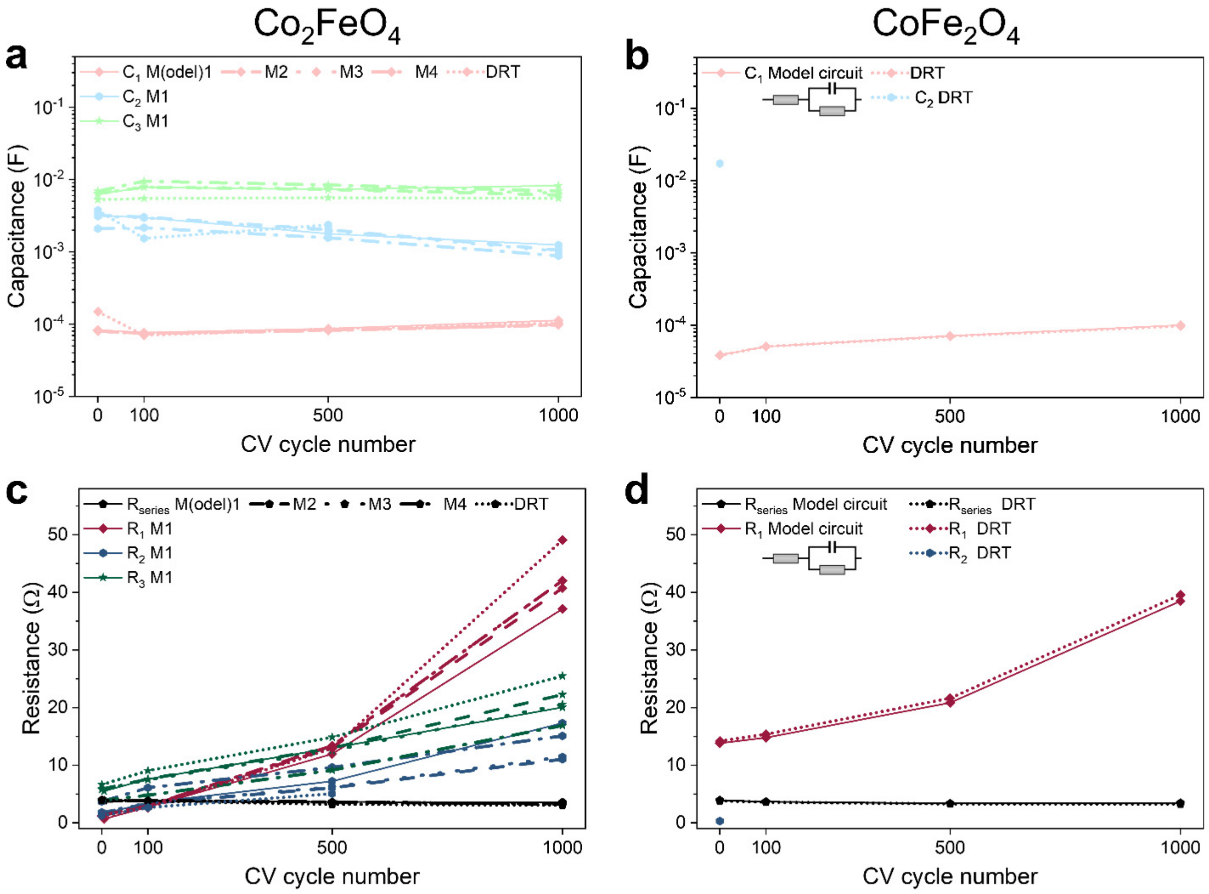


**Supplementary Fig. 25 Capacitances and resistances of $Co_2FeO_4$ and $CoFe_2O_4$ during OER.** (**a**,**b**) Capacitances and (**c**,**d**) resistances of $Co_2FeO_4$ and $CoFe_2O_4$ as results of the equivalent circuit fitting using different equivalent circuit models M1 – M4 as shown in Supplementary Fig. 24 (the model circuit shown in inset in (**b**, **d**) for $CoFe_2O_4$ or of the DRT analysis. Detailed description is listed in Supplementary Note 4.

**Supplementary Table 1.** Structure and lattice constants of oxides.

| | Structure | a (Å) | b (Å) | c (Å) |
|---|---|---|---|---|
| $Co_3O_4$ | Cubic, Fd$\bar{3}$m [3] | 8.6 | 8.6 | 8.6 |
| beta-CoOOH | Hexagonal, R$\bar{3}$m [4] | 2.88 | 2.88 | 13.04 |
| $CoO_2$ | Hexagonal, P$\bar{3}$m1 [5] | 2.82 | 2.82 | 4.29 |
| $Fe_2O_3$ | Hexagonal, R$\bar{3}$c [6] | 5.03 | 5.03 | 13.75 |
| beta-FeOOH | Monoclinic,I4/m [7] | 10.55 | 3.03 | 10.57 |

**Supplementary Table 2.** Number of nanoparticles in composition histograms in Fig. 4m-n, Fig. 5m-n and Fig. 6m-n.

| | $Co_2FeO_4$ | | | | $CoFe_2O_4$ | |
|---|---|---|---|---|---|---|
| | Segregated | | Non-segregated | | | |
| No. of nanoparticles | Fe-rich Nanodomains | Co-rich nanodomains | Bulk | O-rich regions | Bulk | O-rich regions |
| Pristine | 26 | 26 | 22 | - | 39 | - |
| 100 cycles | 21 | 21 | 23 | 7 | 28 | - |
| 500 cycles | 17 | 17 | 19 | 18 | 47 | 16 |
| 1000 cycles | 13 | 13 | 22 | 22 | 59 | 17 |

**Supplementary Table 3.** Number of $Co^{II}$ in tetragonal sites in $Co_2FeO_4$ and $CoFe_2O_4$ (highlighted in red) based on the previous work[8].

| Regions | Oxide Stoichiometry | Number of $Co^{II}$ in tetragonal sites |
|---|---|---|
| Co-rich nanodomains of segregated $Co_2FeO_4$ | $Co_{2.1}Fe_{0.9}O_4$ | $[Co^{II}_{0.78}Fe^{III}_{0.22}]^{Td}[Co^{III}_{1.1}Co^{II}_{0.22}Fe^{III}_{0.68}]^{Oh}O_4$ |
| Fe-rich nanodomains of segregated pristine $Co_2FeO_4$ and non-segregated pristine $Co_2FeO_4$ | $Co_{1.7}Fe_{1.3}O_4$ | $[Co^{II}_{0.55}Fe^{III}_{0.45}]^{Td}[Co^{III}_{0.7}Co^{II}_{0.45}Fe^{III}_{0.85}]^{Oh}O_4$ |
| Pristine $CoFe_2O_4$ | $CoFe_2O_4$ | $[Co^{II}_{0.1}Fe^{III}_{0.9}]^{Td}[Co^{II}_{0.9}Fe^{III}_{1.1}]^{Oh}O_4$ |

**Supplementary Note 1 - X-ray absorption spectroscopy (XAS)**

XAS was performed on $Co_2FeO_4$ and $CoFe_2O_4$ in the pristine state and after 1000 cycles, Supplementary Fig. 7. The X-ray absorption near edge structure (XANES) region of Co K-edge of $Co_2FeO_4$ shows a subtle shift towards high energy values after 1000 cycles (Supplementary Fig. 7a), which suggests that the oxidation state of a small volume fraction, potentially only on the surface regions, has increased. Additionally, a gradual decrease in the intensity of the Co pre-edge peak (7709.5 eV) and an increase in the Co white line feature (at ~7729 eV) were observed (Supplementary Fig. 7a, pre-edge region in inset). This result indicates an increase in the octahedrally coordinated Co after 1000 cycles with an accompanying decrease of tetrahedrally coordinated Co according to the previous work[9]. Additionally, nearly no change in the intensity of pre- and post-edge of Fe peaks before and after 1000 cycles of $Co_2FeO_4$ was observed (inset in Supplementary Fig. 7b). By contrast, the Co XAS spectra of $CoFe_2O_4$ shifts to high energy values after 1000 cycles (Supplementary Fig. 7c), indicating the oxidation of Co(II) to Co(III). Furthermore, a reverse change in the intensity of Co and Fe pre- and post-edge feature suggest an increase of octahedrally coordinated Fe and tetrahedrally coordinated Co after 1000 cycles. Thus, $CoFe_2O_4$ may consist of Co(III) and Fe(III) with octahedral coordination at the Fe site after 1000 cycles.

**Supplementary Note 2 – APT specimen preparation**

The glassy carbon electrode deposited with $Co_2FeO_4$ or $CoFe_2O_4$ nanoparticles after CV measurements was covered by a layer of electrodeposited Ni. The adhesion of the glassy carbon electrode to the Ni layer is critical since voids at the interface or within the Ni matrix can present an enormous challenge for APT specimen preparation and data analysis. Therefore, we first examined the quality of adhesion of the electrodeposited layer by preparing a TEM lamella from the cross-section of the glassy carbon. The scanning electron microscope (SEM) image in Supplementary Fig. 11a shows that the electrodeposited Ni film (brighter contrast) has good adhesion to glassy carbon (darker contrast). Additionally, the electrodeposited Ni layer consists of a good distribution of nanoparticles, as shown in the backscattered electron image in Supplementary Fig. 11b. After confirming this, we used the FIB lift-out procedure to prepare the APT specimens[10], Supplementary Figs. 11c-g.

**Supplementary Note 3 – Measurement of oxide stoichiometry of nanoparticles by APT and $H_2$ TPR**

The measurement of oxide stoichiometry by APT strongly depends on the laser pulse energy and associated electric field[11]. Thus, we first investigate the effect of laser energy on the measurement of oxide stoichiometry of the $Co_2FeO_4$ nanoparticles. The APT specimens were prepared by the

procedure described in Supplementary Note 3 and analysed at voltage pulsing mode with a pulse fraction of 20%, and at laser pulsing mode with laser energy of 10, 30 and 60 pJ, respectively (the specimen temperature, pulse frequency and detection rate were kept the same and described in Experimental section). The Co and Fe-containing ions were exported within the (Co+Fe) iso-concentration surfaces at ~21 at.%. Supplementary Fig. 22a shows that the ratios of $Co^{1+}/Co^{2+}$ and $Fe^{1+}/Fe^{2+}$ increase as the laser energy increases, suggesting that higher laser energy yields less secondary ionisation since the electric field decreases. The $O_2^{1+}/O^{1+}$ ratio, in Supplementary Fig. 22b, increases with the laser energy, indicating an enhanced complex ion generation at higher laser energy. Additionally, the O concentration measured at voltage pulsing mode is 40.1 ± 0.4 at.%, and the value increases with laser energy until it reaches a plateau of ~ 46.0 at.% at 30 pJ (Supplementary Fig. 22c). Thus, the oxygen concentration measured by APT is lower than the nominal oxide stoichiometry of $Co_2FeO_4$. The oxygen deficiency may result from multiple hits, e.g.,$^{16}O_2^{2+}$ at 16 Da, since the $^{16}O_2^{2+}$ complex ions most likely evaporate as multiple hits, similar to $^{12}C_2^{2+}$ observed for carbides[12]. To confirm this, the ratio of multiple hits to single hits at 16 Da was plotted in Supplementary Fig. 22d. The ratio is 13.2:1 at voltage pulsing mode, and it drops significantly and reaches a plateau of 1.9:1 after 10 pJ. This result indicates that the proportion of multiple hits at 16 Da decreases as the electric field is lowed. Thus, the increasing O concentration at higher laser energy is possibly the result of the decrease in the proportion of multiple hits at 16 Da. Therefore, a laser pulsing energy of 30 pJ was selected for the APT measurements, so that higher oxygen deficiency by multiple hits at lower laser energy and a higher number of complex ions at higher laser energy can be balanced.

To compare the values measured by APT with the actual oxygen content, we conducted the $H_2$-temperature programmed reduction ($H_2$ TPR) measurement. A reference measurement was first performed on CuO. Specifically, 0.1069 g of Cu was heated to 450 °C with a heating rate of 5 °C $min^{-1}$ in 84.1 Nml $min^{-1}$ 4.58 % $H_2$/Ar. We can calculate the ratio of oxygen to metal (O/Metal) by using the following equation:

$$\frac{O}{Metal} = \frac{n_O}{n_{Metal}} = \frac{n_O \times M_{metal}}{m - n_O \times M_O}$$

Where $n_O$ and $n_{Metal}$ are the molar quantity of oxygen and metal, $M_O$ is the molar mass of oxygen, m is the weight of metal oxide sample.

The consumption of $H_2$ ($n_{H2}$, mol) is consistent with the O atom quantity ($n_o = n_{H2}$) in the measured sample, calculated by the integral of the $H_2$ TPR curve (grey curve in Supplementary Fig. 23). The weight of the measured sample and total consumption of $H_2$ is 0.1069 g and 0.00135 mol, respectively. Thus, the O/Cu atomic ratio is 1.01, suggesting an oxide stoichiometry of $CuO_{1.01}$ for

CuO. Afterwards, we performed $H_2$ TPR measurement on the pristine $Co_2FeO_4$ nanoparticles. The weight of $Co_2FeO_4$ and $H_2$ consumption is 0.1166 g and 0.00182 mol, respectively (blue curve in Supplementary Fig. 23). The molar mass of $Co_2FeO_4$ is estimated to be ~ 57.7 g/mol, which yields the O/(Co+Fe) ratio of 1.2 (the ratio of Co/Fe (~1.38) can be obtained from APT data). The O/(Co+Fe) ratio measured by APT is ~0.82, 1.46 times lower than the value measured by $H_2$ TPR. In this study, we used this correction factor (1.46) to 'calibrate' the oxygen counts, as the proportion of multiple hits ($^{16}O_2^{2+}$) that causes the deficiency in oxygen content shall be similar when the same operating conditions were used for APT measurements.

**Supplementary Note 4 – Electrochemical impedance spectroscopy (EIS)**

The impedance data were checked and processed regarding artefacts violating causality and stationarity by applying a linear Kramers-Kronig test[13] and a ZHIT algorithm[14]. The distribution of relaxation times (DRT) was determined by a deconvolution approach[15] based on radial basis functions (RBF) with a discretisation factor of $10^{-4}$ and terms proportional to the second derivative of RBF included as extra penalty in the sum of squares minimisation. To calculate corresponding resistance and capacitance values, the DRT function $\gamma(\ln\tau)$ was fitted to estimate peak areas and centres using OriginPro 2019 (OriginLab). Equivalent circuit fitting was performed by applying suitable weighting modes with different model circuits, as displayed in Supplementary Fig. 24. All fitting errors were below 7 %.
DRT was further analysed to determine the number of distinguishable electrochemical sub-processes (regarding their characteristic time constant $\tau = R \cdot C$). For deconvoluting DRTs, the impedance of an equivalent circuit consisting of ohmic resistance, and an infinite series of parallel resistors and capacitors (Voigt circuit) is fitted to the experimentally obtained impedance data. The distribution function of time constants describing the experimental system was determined without presuming a certain number of parallel resistor and capacitor (RC) elements in series. The applied software uses a regularised regression approach applying radial basis functions[15] to solve the respective intrinsically ill-posed problem.

The DRT of $Co_2FeO_4$ (Fig. 7a in the main text, inset) shows three distinct peaks for the pristine nanoparticles as well as after 100 and 500 cycles. After 1000 cycles, the signal of the electrochemical process related to the low-τ-region (which corresponds to the signal appearing in the high-frequency region of the complex plane plot) overlaps with the mid-τ-region signal due to a pronounced increase in the resistance. By contrast, the inverse spinel $CoFe_2O_4$ features only one peak showing increasing resistance for higher cycle numbers and a corresponding shift to higher time constants.

Based on the DRT analysis, equivalent circuit fitting was performed with the circuit models given

in Fig. 7c, d (main text), yielding the respective resistances and capacitances plotted for the pristine material and after 100, 500 and 1000 cycles. They agree well with the values obtained from the DRT results (Supplementary Fig. 25). For $Co_2FeO_4$, four different arrangements of three parallel RC elements (Supplementary Fig. 24) were used to fit the experimental impedance data. As seen in Supplementary Fig. 25, the determined capacitances and resistances do not significantly differ for the considered equivalent circuit models. In conclusion, the trends observed for the identified frequency-dependent electrochemical processes during cyclic voltammetry are considered solid, although the experimental data can be described by different models of three RC elements and one ohmic resistor.

The equivalent circuit model for $CoFe_2O_4$ consists of an ohmic resistor, mainly characterised by the electrolyte resistance between working and reference electrode, in series to a RC element. This is interpreted as an electric double layer capacitor in parallel to a resistor of the faradaic resistance for the OER. Although the OER mechanism comprises several electron transfer steps, these can cause only one semicircle in the complex plane plot (Fig. 7b) since the respective serial resistances are in parallel to the same capacitor. Thus, a faradaic resistance is determined, which corresponds to the sum reaction (expected to mainly account for the rate-determining reaction step).

Furthermore, the three processes with different time constants observed for $Co_2FeO_4$ (inset, Fig. 7a) confirm that additional capacitive material characteristics must be present besides electric double layer capacitance. These electrochemical capacitive properties are likely related to pseudocapacitance which arises due to fast faradaic transformation reactions of mixed oxide materials in the near-surface region, as has been known for $Co_3O_4$[16]. The capacitances $C_2$ and $C_3$ for $Co_2FeO_4$ are two orders of magnitude larger than $C_1$ for which a double layer capacitance is assigned (Fig. 7a). The pre-OER potential range of the cyclic voltammograms (Fig. 1c, main text) also shows currents exceeding expected values for double layer charging. Furthermore, $R_1$ increases with increasing number of CV cycles, in particular, after 500 and 1000 cycles. Thus, the kinetics of $OH^-$adsorption and reversible Co oxide oxidation become increasingly hindered, shifting and diminishing CV features and decreasing the OER activity (Fig. 1, main text). The resistance $R_3$ of the $O_2$-formation step increases less significantly than $R_1$, which suggests a fast consumption of adsorbed oxygen species and an absence of OH-groups for $(Co,Fe)O_2$.

## Supplementary References


1 Wu, G., Wang, J., Ding, W., Nie, Y., Li, L., Qi, X., Chen, S. & Wei, Z. A strategy to promote the electrocatalytic activity of spinels for oxygen reduction by structure reversal. *Angewandte Chemie International Edition* **55**, 1340-1344 (2016).

2 Bergmann, A., Martinez-Moreno, E., Teschner, D., Chernev, P., Gliech, M., de Araujo, J. F., Reier, T., Dau, H. & Strasser, P. Reversible amorphization and the catalytically active state of crystalline Co3O4 during oxygen evolution. *Nature Communication* **6**, 8625, doi:10.1038/ncomms9625 (2015).

3 Smith, W. & Hobson, A. The structure of cobalt oxide, Co3O4. *Acta Crystallographica Section B: Structural Crystallography and Crystal Chemistry* **29**, 362-363 (1973).

4 Delaplane, R. G., Ibers, J. A., Ferraro, J. R. & Rush, J. J. Diffraction and spectroscopic studies of the cobaltic acid system HCoC2–DCoO2. *The Journal of Chemical Physics* **50**, 1920-1927 (1969).

5 Amatucci, G., Tarascon, J. & Klein, L. CoO2, the end member of the Li x CoO2 solid solution. *Journal of The Electrochemical Society* **143**, 1114 (1996).

6 Finger, L. W. & Hazen, R. M. Crystal structure and isothermal compression of Fe2O3, Cr2O3, and V2O3 to 50 kbars. *Journal of Applied Physics* **51**, 5362-5367 (1980).

7 Post, J. E. & Buchwald, V. F. Crystal structure refinement of akaganeite. *American Mineralogist* **76**, 272-277 (1991).

8 Jung, I.-H., Decterov, S. A., Pelton, A. D., Kim, H.-M. & Kang, Y.-B. Thermodynamic evaluation and modeling of the Fe–Co–O system. *Acta Materialia* **52**, 507-519 (2004).

9 De Groot, F., Vankó, G. & Glatzel, P. The 1s x-ray absorption pre-edge structures in transition metal oxides. *Journal of Physics: Condensed Matter* **21**, 104207 (2009).

10 Thompson, K., Lawrence, D., Larson, D., Olson, J., Kelly, T. & Gorman, B. In situ site-specific specimen preparation for atom probe tomography. *Ultramicroscopy* **107**, 131-139 (2007).

11 Devaraj, A., Colby, R., Hess, W. P., Perea, D. E. & Thevuthasan, S. Role of photoexcitation and field ionization in the measurement of accurate oxide stoichiometry by laser-assisted atom probe tomography. *The journal of physical chemistry letters* **4**, 993-998 (2013).

12 Marceau, R. K., Choi, P. & Raabe, D. Understanding the detection of carbon in austenitic high-Mn steel using atom probe tomography. *Ultramicroscopy* **132**, 239-247 (2013).

13 Boukamp, B. A. A linear Kronig-Kramers transform test for immittance data validation. *Journal of the electrochemical society* **142**, 1885 (1995).

14 Schiller, C., Richter, F., Gülzow, E. & Wagner, N. Validation and evaluation of electrochemical impedance spectra of systems with states that change with time. *Physical Chemistry Chemical Physics* **3**, 374-378 (2001).

15 Wan, T. H., Saccoccio, M., Chen, C. & Ciucci, F. Influence of the discretization methods on the distribution of relaxation times deconvolution: implementing radial basis functions with DRTtools. *Electrochimica Acta* **184**, 483-499 (2015).

16 Liu, T.-C., Pell, W. & Conway, B. Stages in the development of thick cobalt oxide films exhibiting reversible redox behavior and pseudocapacitance. *Electrochimica acta* **44**, 2829-2842 (1999).
